# Supplementary material for: Dementia in UK South Asians: a scoping review of the literature
Source: BMJ Open. 2018 Apr 12;8(4):e020290. doi: 10.1136/bmjopen-2017-020290 (PMC5898329; doi:10.1136/bmjopen-2017-020290)
Supplement: Supplementary data [file bmjopen-2017-020290supp001.pdf]

Ovid Medline(R) 1946 to April Week 2 2016

View Saved

Search History (9)

	# ▲	Searches	Results	Type	
	1	Dementia/ or dementia.mp.	84164	Advanced	More
	2	Alzheimer Disease/ or Alzheimer* disease.mp.	101182	Advanced	More
	3	1 or 2	155203	Advanced	More
	4	ethnic*.mp.	126694	Advanced	More
	5	Asian.mp. or Asian Continental Ancestry Group/	89665	Advanced	More
	6	black.mp. or African Continental Ancestry Group/	104349	Advanced	More
	7	Ethnic Groups/ or multiethnic.mp.	54225	Advanced	More
	8	4 or 5 or 6 or 7	288052	Advanced	More

☐	9	3 and 8	2556	Advanced
