# Supplementary material for: Dementia in UK South Asians: a scoping review of the literature
Source: BMJ Open. 2018 Apr 12;8(4):e020290. doi: 10.1136/bmjopen-2017-020290 (PMC5898329; doi:10.1136/bmjopen-2017-020290)
Supplement: Author's manuscript [file bmjopen-2017-020290.draft_revisions.pdf]

BMJ Open

BMJ Open is committed to open peer review. As part of this commitment we make the peer review history of every article we publish publicly available.

When an article is published we post the peer reviewers' comments and the authors' responses online. We also post the versions of the paper that were used during peer review. These are the versions that the peer review comments apply to.

The versions of the paper that follow are the versions that were submitted during the peer review process. They are not the versions of record or the final published versions. They should not be cited or distributed as the published version of this manuscript.

BMJ Open is an open access journal and the full, final, typeset and author-corrected version of record of the manuscript is available on our site with no access controls, subscription charges or pay-per-view fees (http://bmjopen.bmj.com).

If you have any questions on BMJ Open's open peer review process please email info.bmjopen@bmj.com

Dementia in UK South Asians: A scoping review of the literature

Journal:	*BMJ Open*
Manuscript ID	bmjopen-2017-020290
Article Type:	Research
Date Submitted by the Author:	26-Oct-2017
Complete List of Authors:	Blakemore, Amy; University of Manchester, Division of Nursing, Social Work and Midwifery, School of Health Sciences, Manchester Academic Health Science Centre<br>Kenning, Cassandra; University of Manchester, Division of Population Health, Health Services Research and Primary Care, School of Health Sciences, Manchester Academic Health Science Centre<br>Mirza, Nadine; University of Manchester, Division of Population Health, Health Services Research and Primary Care, School of Health Sciences, Manchester Academic Health Science Centre<br>Daker-White, Gavin; University of Manchester, NIHR Greater Manchester Primary Care Patient Safety Translational Research Centre, Manchester Academic Health Science Centre<br>Panagioti, Maria; University of Manchester, Division of Population Health, Health Services Research and Primary Care, School of Health Sciences, Manchester Academic Health Science Centre<br>Waheed, Waquas; University of Manchester, Division of Population Health, Health Services Research and Primary Care, School of Health Sciences, Manchester Academic Health Science Centre
**Primary Subject Heading**:	Health services research
Secondary Subject Heading:	Neurology
Keywords:	Dementia < NEUROLOGY, South Asian, United Kingdom, Scoping Review

SCHOLARONE™ Manuscripts

Dementia in UK South Asians: A scoping review of the literature

Running head: Dementia in UK South Asians

Key Words: Dementia, United Kingdom, South Asian, Scoping Review

Authors:

Dr Amy Blakemore
Division of Nursing, Social Work and Midwifery, School of Health Sciences, Faculty of Biology, Medicine and Health, The University of Manchester, Manchester Academic Health Science Centre, Manchester, United Kingdom

Dr Cassandra Kenning
Division of Population Health, Health Services Research and Primary Care, School of Health Sciences, The University of Manchester, Manchester Academic Health Science Centre, Manchester, Manchester, United Kingdom

Miss Nadine Mirza
Division of Population Health, Health Services Research and Primary Care, School of Health Sciences, The University of Manchester, Manchester Academic Health Science Centre, Manchester, United Kingdom

Dr Gavin Daker-White
NIHR Greater Manchester Primary Care Patient Safety Translational Research Centre, The University of Manchester, Manchester Academic Health Science Centre, Manchester, United Kingdom

Dr Maria Panagioti
Division of Population Health, Health Services Research and Primary Care, School of Health Sciences, The University of Manchester, Manchester Academic Health Science Centre, Manchester, United Kingdom

Dr Waquas Waheed
Division of Population Health, Health Services Research and Primary Care, School of Health Sciences, The University of Manchester, Manchester Academic Health Science Centre, Manchester, United Kingdom

Corresponding Author
Dr Amy Blakemore, Division of Nursing, Social Work and Midwifery, Jean McFarlane Building, Oxford Road, The University of Manchester, Manchester M13 9PL
Tel: 0161 306 7846
Email: amy.blakemore@manchester.ac.uk

Word Count: 4331

For peer review only - http://bmjopen.bmj.com/site/about/guidelines.xhtml

Abstract (298 Words)

Objective

Over 850,000 people live with dementia in the United Kingdom (UK). A proportion of these people will be South Asians, who make up over 5% of the total UK population. Little is known about the prevalence, experience, and treatment of dementia in the UK South Asian population. The aim of this scoping review is to identify and summarise dementia studies conducted in the UK South Asian population to identify the gaps in the literature which should be addressed in future research.

Method

Databases were systematically searched using a comprehensive search strategy to identify studies. The methodological framework for conducting scoping reviews was followed. An extraction form was developed to chart data and collate study characteristics and findings. Studies were then grouped into six categories: prevalence and characteristics; diagnosis validation and screening; knowledge, understanding and attitudes; help-seeking; experience of dementia; service organisation and delivery.

Results

A total of 6481 studies were identified and screened. Twenty six studies were eligible for inclusion in the scoping review. Studies of prevalence, diagnosis, and service organisation and delivery are limited. We did not find any clinical trials of culturally appropriate interventions for South Asians with dementia in the UK. The existing evidence comes from small scale service evaluations and case studies.

Conclusions

This is the first scoping review of the literature to identify priority areas for research to improve care for UK South Asians with dementia. Future research should first focus on developing and validating culturally appropriate diagnostic tools for UK South Asians and then conducting high quality epidemiological studies in order to accurately identify the prevalence of dementia in this group. The cultural adaptation of interventions for dementia

For peer review only
For peer review only - http://bmjopen.bmj.com/site/about/guidelines.xhtml

and testing in randomised controlled trials is also vital to ensure that there are appropriate treatments available for UK South Asians to access.

Strengths and Limitations of the Study

- The first scoping review of the literature to identify priority areas for future research and care of UK South Asians with dementia.
- Comprehensive and systematic search which was conducted in line with published guidelines for scoping reviews.
- A comprehensive and broad search strategy was developed however, a lack of standardisation around some key search terms could mean some studies were not identified.
- This review is not able to provide a definitive answer to any of the key themes discussed but its purpose is to map the literature and provide a much needed framework to guide future research directions for UK south Asians with dementia.

For peer review only

For peer review only - http://bmjopen.bmj.com/site/about/guidelines.xhtml

Introduction

As the global population ages the prevalence of dementia increases; there are an estimated 47 million people living with dementia worldwide and this figure is set to increase to over 75.6 million by 2030.[1,2] In the United Kingdom (UK) alone there are over 850,000 people living with dementia at a cost of £26 billion per year.[1] The rising prevalence of dementia and its associated cost and burden has generated an increased focus on the timely screening, diagnosis and treatment of dementia.

Ethnic minority groups make up over 14% of the total UK population, many of whom are South Asian (Pakistan, India, and Bangladesh). In the 2001 census South Asians made up 3.9% of the total UK population and by 2011 this figure had risen to 5.3%.[3] It is estimated that there are over 25,000 people from ethnic minority groups living with dementia in the UK.[4] However, the true prevalence of dementia within the UK South Asian community is yet to be established.

There are potential difficulties in identifying, diagnosing, and treating dementia in UK South Asian population. In the South Asian community only 35% of older people can speak English and only 21% can read and write English, with most relying on their first language which for many is Urdu.[3,5] There is also lack of awareness of dementia within the South Asian community with many people viewing memory loss as a normal part of ageing or understanding it using religious beliefs.[6] For those who do seek help there is a lack of culturally appropriate services and accurately translated neuropsychological assessments for UK South Asians.[7]

The aim of this scoping review is identify and summarise dementia studies conducted in the UK South Asian population in order to identify gaps in the literature which need to be addressed in future research.

For peer review only

Methods

A scoping review was conducted in accordance with the methodological framework for scoping reviews published by Arkskey and O'Malley (2007) and further developed by Levac et al. (2010).[8,9] The framework includes guidance on the following areas: identifying the research question, searching for relevant studies, selecting studies, charting the data, and collating and summarising results.

Inclusion criteria and exclusion criteria

Studies that had included UK South Asian dementia patients were eligible for inclusion, as well as those that had focused on family carers, and healthcare professionals working with dementia patients. We did not exclude studies based on methodology or year of publication. Published dissertations were included but any unpublished dissertation was not. Systematic reviews and narrative literature reviews were not included but reference lists were searched to identity additional primary studies for inclusion. Conference proceedings were not included.

We excluded studies that did not report on South Asian participants alone, as a comparator group, or where data could not be separated from data for participants of other ethnicities. We excluded studies that were not published in the English language.

Search strategy

Search terms were kept broad to identify the maximum number of studies which were eligible for inclusion. The search strategy was developed within the team to identify all studies relevant to ethnicity and dementia, this was then refined through hand checking to those which included UK South Asian participants. Search terms included: Dementia, Alzheimer* Disease; ethnic*, Asian, Black, African, minority, ethnic group, multi-ethnic. See additional material 1 for the full search strategy.

The search was conducted without a study design filter in order to retrieve studies using all methodologies, including: systematic reviews, qualitative studies, quantitative studies, case studies, and service evaluations.

For peer review only - http://bmjopen.bmj.com/site/about/guidelines.xhtml

The following databases were searched: Cochrane Register of Controlled Trials (CENTRAL), MEDLINE (OVID), PsycINFO (Ovid), Embase (Ovid), Cochrane database of systematic Reviews. The search was initially conducted in April 2016 and updated in June 2017. In addition to the database searches, we hand searched the reference lists of all relevant systematic reviews and literature reviews that were returned in the search to identify any additional papers for inclusion.

Screening and charting the data

Electronic search results were managed using EndNote X7 and Microsoft Excel. Titles and abstracts of all citations were first screened by author (AB), those that were not related to dementia and South Asians, or had been conducted outside of the UK, were discarded. The full text of all potentially eligible papers were then obtained and assessed against the inclusion criteria (AB and WW). Where the full text of papers was not available authors were contacted to request the paper. Any ambiguities about whether or not a study met the inclusion criteria were resolved by discussion at a meeting attended by all authors.

Studies that met the review criteria were charted using a data extraction sheet designed by AB and WW. Data were primarily extracted by AB and reviewed by WW. Data was extracted for the following domains: year of publication, study design, ethnicity, aim of study, study setting (community/primary care/hospital), eligibility criteria, participants (patient/carer/healthcare staff), type of dementia, age of participants, sample size, main findings.

Data synthesis

Studies were grouped into categories according to their primary focus (AB, CK, WW): prevalence and characteristics; service organisation and delivery; views and experiences; validation, screening and diagnosis. All data are reported in a narrative format. As this is a scoping review there is no formal review of the quality of the included literature.[8,9]

For peer review only - http://bmjopen.bmj.com/site/about/guidelines.xhtml

Results

Description of included studies

The initial search identified 9085 studies, 6481 after duplicates were removed. Screening of the titles and abstracts left 120 studies that met the criteria for full text review, 25 were then selected for inclusion.[10-34] To update the review the search was rerun in June 2017; 1 new paper was found to be eligible for inclusion, bringing the total number of included studies to 26.[35] Figure 1 shows the flow of study selection and numbers of excluded studies with reasons.[36]

Table 1 shows a summary of the findings for all 25 included studies. For the purpose of narrative review we grouped the studies into six categories: prevalence and characteristics; diagnosis validation and screening; knowledge, understanding and attitudes; help-seeking; experience of dementia; service organisation and delivery.

Prevalence and Characteristics

We identified four articles that had looked at either the prevalence or characteristics of dementia in UK South Asians.[12,15,25,33]

Bhatnagar (1997) looked at the prevalence of dementia in a community sample of people from the Indian subcontinent living in Bradford, UK.[12] Diagnostic interviews were conducted with 100 participants and the prevalence of dementia was reported as 4%. However, the reliability of the diagnosis when tested using a Hindi translation of the Geriatric Mental State (GMS-A), which found a prevalence of 7%, was poor. The authors suggest that this is due to a lack of cultural adaptation of the measure. For example some interviewees would have little understanding of western calendar months, a knowledge of which forms part of the screening measure.[12]

Two articles reported characteristics of ethnic elders from the Indian subcontinent living in west London and attending a psychogeriatric service.[25,33] Both published reports were from the same sample population, the first consisting of cross sectional data from a census conducted in August 1995,[33] and the second a two year study of all new cases entering the

For peer review only - http://bmjopen.bmj.com/site/about/guidelines.xhtml

For peer review only

service between August 1995 and July 1997.[25] Sample sizes were small with South Asians making up only 12%[25] and 17%[33] of the total number of recruited participants. Redelinghuys et al. 1997[33] found that only a small subsample of participants had dementia, with no significant difference found between those from the Indian subcontinent (n=6, 15%) and white British elders (n=43, 22%). However, Odutoye et al. 1999 [24] reported that over that time period there was a significant difference in the incidence of dementia with elders from the Indian subcontinent being less likely to have a dementia.

Haider et al. (2004) conducted a study of the behavioural and psychological signs and symptoms of dementia using the Behavioural Pathology in Alzheimer's disease Rating Scale (BEHAVE-AD) rating scale.[15,37] They compared a sample of patients from the Indian subcontinent with a White British sample living in West London. They did not find any major differences between the two groups.

In summary studies have attempted to identify the prevalence of dementia in South Asians living in the UK. Most have reported that there is no difference in the prevalence between South Asian and White British participants. However, sample sizes in these studies have been unanimously small and the studies are over 14 years old, therefore it is difficult draw conclusions about the definitive prevalence.

Diagnosis, Validation & Screening

Three papers were identified that discussed diagnosis of dementia in UK South Asians or validation of screening tools. Firstly, Shah et al. (1998) investigated the stability of dementia diagnosis over time in a Gujarati population in Leicester using a Gujarati version of the Mini-Mental State (MMSE) examination.[29] They reported that the diagnosis of dementia was stable at follow-up over a period of more than 2 years (26-32 months) which indicates that the measure has good test-retest reliability in this population.

Secondly, two studies were identified which had looked to validate the MMSE dementia screening instrument for use in the UK South Asian population. Lindesay et al. (1997) investigated whether a Gujarati version of the MMSE could be used as a screening instrument.[22] They reported that the MMSE performed well in the identification of

For peer review only - http://bmjopen.bmj.com/site/about/guidelines.xhtml

moderate to severe dementia but was less effective in detecting cases of mild dementia in the Gujarati group when compared to a group of White British participants. Similarly, Rait et al. (2000) tested the MMSE in a population of South Asians, including Gujarati and Pakistani participants.[27] They found high levels of sensitivity and specificity for the MMSE but with a lower cut-off score for the identification of mild dementia in the South Asian group.

Findings indicate that the Guajarati version of the MMSE may be effective at identifying dementia, especially where the diagnosis is certain and therefore symptoms are moderate to severe. However, it may be less effective in identifying dementia where symptoms are mild or diagnosis is uncertain.

Knowledge, Understanding, and Attitudes

We identified six studies which had set out to understand knowledge, understanding and attitudes to dementia in the UK South Asian community. Knowledge about dementia in Indian (n=91) and White UK/Irish/European (n=55) people was assessed using the Dementia Knowledge Questionnaire (DKQ) by Purandare et al.[26] They found that both groups had poor knowledge of causes and symptoms, and Indian older people had significantly less 'basic knowledge' about dementia. Here the authors define 'basic knowledge as including knowledge of epidemiology, aetiology, and symptomatology as measured by the Dementia Knowledge Questionnaire (DKQ).[38] A further series of focus groups investigated general population knowledge of dementia with 28 Sikh participants.[31] Again they found poor knowledge of dementia and that Sikh participants placed greater emphasis on physical illnesses than disorders such as dementia. La Fontaine held focus groups with a population sample of 49 Punjabi Indian participants.[19] One of their key findings was the failure of participants to directly refer to dementia or even to use lay terms to discuss it which the authors conclude indicates a lack of recognition of dementia as a concept.

Turner et al. (2005) explored knowledge and attitudes to dementia in a qualitative community study of South Asian (n=96) and White older people (n=96) and also found reduced knowledge of dementia in the South Asian group. Furthermore, their findings highlighted differences in attitudes to dementia care, with the South Asian group reporting

For peer review only - http://bmjopen.bmj.com/site/about/guidelines.xhtml

that care should be provided by family and friends.[32] In interviews with 10 South Asian family carers Lawrence et al. (2008) looked at traditional versus non-traditional care giver ideology.[20] The majority of South Asian carers possessed a traditional ideology in that they saw care giving as natural, expected, and virtuous. However, where understanding of dementia is poor problems can occur within the family caring relationship due to beliefs about the causes of dementia in the South Asian community and the attribution of blame on to the person with dementia.[10]

Adamson et al. (2005) interviewed 15 South Asian carers living in five cities across the UK.[10] The study looked at the relationship between individual experiences, cultural factors and social structures within this minority population. Carers tended to interpret their caring roles as an expected part of their cultural heritage and a continuation of their family relationships. They also likened the experiences of this group to other chronic illness care and suggest that the understanding of the experiences of chronic care may be useful in understanding experiences in informal care for dementia.

In summary, several studies have explored the knowledge of dementia and beliefs and attitudes to care in UK South Asians. All report that knowledge is poor and this group are often more focussed on physical illness rather than conditions such as dementia. Careis preferred to be provided by family and within the community and therefore, UK South Asians may not access formal healthcare for dementia. Giebel et al. (2016) have adapted the Barts Explanatory Model Inventory Checklist (BEMI-C) for use with South Asian ethnic minority groups (BEMI-Dementia).[35] They conducted 25 qualitative interviews with South Asians and identified 123 new perceptions around their understanding of dementia. These were added to the BEMI-C to create a new checklist (BEMI-D) to better assess the barriers to dementia service uptake in this group.

Help-seeking

Five papers were identified that focused on different aspects of help-seeking for dementia in the UK South Asian population.

For peer review only - http://bmjopen.bmj.com/site/about/guidelines.xhtml

Two studies had looked at barriers to help-seeking for dementia. Firstly, in a community sample of English or Bengali speaking UK South Asians who did not have a diagnosis of dementia Mukadam et al (2015) identified the barriers and facilitators to help-seeking for memory problems.[24] They identified four main categories of barriers which interacted to prevent timely diagnosis of dementia: barriers to help-seeking for memory problems; the threshold for seeking help for memory problems; ways to overcome barriers to help-seeking; what features an educational resource should have. Secondly, Hailstone et al. (2016) devised and validated a theory of planned behaviour (Attitudes of People from Ethnic Minorities for Help-seeking for Dementia: APEND) questionnaire to predict medical help-seeking for dementia in UK South Asians.[16] They found that attitudes to dementia predicted 77% of variance in help-seeking with the strongest predictor being perceived social pressure.

Furthermore, there were two studies which had specifically identified religious explanations as barriers to help-seeking for dementia in South Asians. Mackenzie et al. (2006) interviewed 11 Pakistani and 5 Indian carers and found that stigma resulted from religious and magical beliefs around the causes of dementia and resulted in concealment from their own community and delays in help-seeking.[23] Giebel et al. (2016) looked at the differences in the perceptions of South Asians who do and do not consult a General Practitioner (GP) about dementia.[14] They found those who did not consult a GP were significantly more likely to consider memory problems as given by god, with the view that medical intervention was therefore inappropriate. In a case study of a Muslim, Pakistani patient accessing healthcare for dementia in the UK, Regan et al. (2016) further highlight the importance of understanding a person's religious community and its role in providing both support and reducing stigma and isolation.[34]

The included studies highlight that attitudes and beliefs about dementia can serve as barriers to accessing healthcare, in particular understanding dementia within a religious context can delay help seeking. However, religious communities can also play a vital role in supporting patients with dementia and in reducing stigma. This may be the case across other ethnic minority communities in the UK, not just South Asians. It is therefore important to learn more about the religious context in which people understand dementia and for

For peer review only - http://bmjopen.bmj.com/site/about/guidelines.xhtml

healthcare providers to engage with local communities and religious leaders in order to work in partnership with them to increase support and reduce stigma.

Experiences of Dementia

We identified three articles that looked at understanding the experience of dementia for UK South Asians. Bowes et al 2003 explored the experiences of South Asian patients with dementia in Scotland.[13] Interviews were conducted with 11 health professionals, and four case studies were built up through multiple interviews with 4 people with dementia, as well as interviews with their family and carers. The authors reported overwhelmingly negative experiences of dementia and of health services from the case studies. From the interviews with health professionals the authors concluded that there was a need to develop and promote culturally sensitive services. Furthermore, the article by Jutlla (2015) looked at the experiences of 12 Sikh carers, caring for a family member with dementia, in Wolverhampton.[17] In particular they looked at the influences of migration experiences and migration identities. They found that knowledge a person's background and experiences is important for understanding how they then experience health services and caring roles. This is presented as an advocate, not just for culturally appropriate services, but also for person-centred dementia care.

Lawrence et al. (2011) looked at experiences of dementia in three ethnic groups, 11 Black Caribbean, 9 South Asian and 10 White British older people with dementia.[21] The paper reported a comparison in the personal experiences of the condition in the three groups. They reported similar themes across groups with a key finding being that patients assessed their condition by the degree to which it interfered with 'valued elements of life'. The authors concluded that development of culturally sensitive approaches to care should promote roles, relationships and activities that the patient values.

Service Organisation and Delivery

We found three articles that discussed service organisation and delivery for South Asian patients with dementia in the UK. Firstly, in 1999 Shah, a Gujarati Psychogeriatrician, published a descriptive account of his own experiences working with 12 Gujarati patients in

For peer review only - http://bmjopen.bmj.com/site/about/guidelines.xhtml

their language.[30] Four of the patients had a diagnosis of dementia. He identified particular problems in eliciting cognitive symptoms due to problems with the translation and the lack of culturally appropriate assessments.[30] Secondly, Kaur et al. (2010) writes about the role an Asian link nurse for Punjabi speaking people of Asian origin in a dementia service in Wolverhampton.[18] The authors reported success in providing appropriate and culturally sensitive help and information for healthcare professionals, voluntary services, and South Asian patients with dementia. Thirdly, Seabrooke and Milne (2009) discuss the Dementia Collaborative Project in North West Kent which aimed to raise awareness of memory problems in South Asians and facilitate access to screening and diagnosis.[28] The authors reported an increase in referrals to a specialist clinic for memory assessments, some of which were for South Asian patients, and an increase in health professional's knowledge of memory problems in South Asians.

For peer review only

Discussion

This review has scoped and synthesised the available literature on dementia in the UK South Asian population. We identified 26 studies which we found focussed on prevalence, diagnosis and screening, knowledge and attitudes, help seeking, experience, and service organisation and delivery. Overall, this review highlight that research on dementia in UK South Asians is limited.

There have been few studies of the prevalence of dementia within UK South Asians and where studies have set out to identify prevalence, sample sizes have been small. Furthermore, studies of dementia prevalence in South Asians are old, with the most recent being published in 2004.[15] There is a need for large epidemiological studies of dementia in UK South Asians in order to confirm the prevalence nationally. There is also a need for studies embedded within epidemiological work that can explore the current experience of South Asians with dementia in the context of dementia healthcare.

We found only two studies that aimed to validate the MMSE screening tool within the UK South Asian population.[22,27] These two studies limited South Asians to a screening process with no access to receiving a confirmed diagnosis. We did not identify any studies that had addressed the introduction and validation of a purely diagnostic assessment for UK South Asians, such as the Addenbrooke's Cognitive Examination Version III or the Motreal Cognitive Assessment.[39,40] This compromises current knowledge of dementia prevalence within in UK South Asians, as without a culturally appropriate, validated diagnostic assessment individuals cannot be diagnosed. The lack of a culturally adapted and validated diagnostic tool risks that UK South Asians who enter into the diagnostic process will receive higher rates of false positive or false negative scores. This invalidates the current diagnoses given to UK South Asians.

Most of the existing research has been conducted in the community which reflects the fact that UK South Asians do not often access formal healthcare services for memory problems. We need to develop culturally sensitive screening tools, diagnostic tests, and interventions whilst also engaging communities. It is important to develop interventions in parallel with

For peer review only - http://bmjopen.bmj.com/site/about/guidelines.xhtml

community engagement to ensure that there are culturally appropriate services ready to be accessed if engagement is successfully increased.[6]

We were unable to identify a single clinical trial of an intervention for dementia in the UK South Asian population, either amongst patients or carers. This highlights a critical gap in ongoing dementia research and indicates a neglect of 5.3% of the UK population that identify as South Asian and calls into question whether it is appropriate to implement existing interventions for this ethnic group without any assessment of their feasibility, acceptability, and effectiveness.[3] This review has identified a number of different views and perceptions held about dementia by UK South Asians. Therefore, existing interventions need to be adapted or new interventions which can accommodate for these differences need to be developed for this particular population.

This review has highlighted factors which impact on our ability to conduct intervention trials in this population; these may include issues surrounding diagnosis and the lack of appropriately validated diagnostic instruments, as well as attitudes and beliefs about dementia which may affect recruitment. Much of the qualitative work identified in this review has highlighted the religious explanations by which the South Asian community often understand symptoms of dementia. Studies report that this causes a barrier to help seeking and will result in delayed treatment. Furthermore, due to religious explanatory models for dementia patients and family carers often perceive treatment as inappropriate. Culturally sensitive community engagement work is needed to engage the South Asian community and encourage understanding of dementia. New interventions should also acknowledge and include the family approach to care which is seen as of paramount importance in the South Asian community.

We suggest that undertaking the cultural adaptation and validation of diagnostic assessments within UK South Asians would improve the diagnostic process. From our findings relating to service organisation and delivery we can see strategies emerging that may increase engagement from this population, such as the ethnic matching of staff and increasing engagement work with South Asian community. These strategies should now be considered when designing clinical trials to test culturally adapted interventions.

For peer review only - http://bmjopen.bmj.com/site/about/guidelines.xhtml

This review is the first review to scope all of the literature on dementia in the UK South Asian population. We scoped the literature in line with the guidelines for scoping reviews and conducted a systematic search of the literature.[9] It is possible that there are other studies that were not identified as part of this search; in part this could be due to a lack of standardisation of the terminologies used in the literature.

The purpose of this review was to map the literature and to provide a useful framework to guide future research directions. It is not designed to provide a definitive answer in any of the key themes discussed because there are important differences across the included studies (e.g. quality, design, and population) which were not extensively evaluated. However, given that this area is under-investigation this scoping review is particularly suited because it can provide a comprehensive account of current progress and challenges which can be used to develop future research and priorities to improve care for UK South Asians.

Conclusion

A number of studies have been published on dementia in the UK South Asian population. However, studies of prevalence, diagnosis, and service organisation and delivery are limited. We found no clinical trials of culturally appropriate interventions for South Asians with dementia in the UK. The existing evidence comes from small scale service evaluations and case studies.

Future research efforts should concentrate on developing and validating culturally appropriate diagnostic tools for UK South Asians. Epidemiological studies are needed to accurately identify the prevalence of dementia in this group. Cultural adaptation and clinical trials of appropriate interventions are needed to run in parallel to diagnosis and community engagement work to ensure there are effectiveness and acceptable treatments for South Asians to access once identified with dementia.

For peer review only - http://bmjopen.bmj.com/site/about/guidelines.xhtml

References

1 Alzheimer's Disease Internataional. *Policy Brief for G8 Heads of Government. The Gloval Impact of Dementia 2013-2050*, <http://www.who.int/mental_health/neurology/dementia/dementia_thematicbrief_epidemiology.pdf> (2013).

2 World Health Organisation. *the Epidemiology and Impact of Dementia: Current state and future trends*, <http://www.who.int/mental_health/neurology/dementia/dementia_thematicbrief_epidemiology.pdf> (2015).

3 Office for National Statistics. *Ethnicity and National Identiity in England and Wales*, <https://www.ons.gov.uk/peoplepopulationandcommunity/culturalidentity/ethnicity/articles/ethnicityandnationalidentityinenglandandwales/2012-12-11> (2012).

4 All-Party Parliamentary Group on Dementia. *Dementia does not discriminate: The experiences of black, Asina and minority ethnic communities*, <https://www.alzheimers.org.uk/download/downloads/id/1857/appg_2013_bame_report.pdf> (2013).

5 Khan, F. & Tadros, G. Complexity in cognitive assessment of elderly British minority ethnic groups: Cultural perspective. *Dementia* **13**, 467-482, doi:doi:10.1177/1471301213475539 (2014).

6 Kenning, C., Daker-White, G., Blakemore, A., Panagioti, M. & Waheed, W. Barriers and facilitators in accessing dementia care by ethnic minority groups: a meta-synthesis of qualitative studies. *BMC Psychiatry* **17**, 316, doi:10.1186/s12888-017-1474-0. (2017).

7 Regan, J. L. Redefining dementia care barriers for ethnic minorities: The religion-culture distinction. *Mental Health, Religion & Culture* **17**, 345-353, doi:http://dx.doi.org/10.1080/13674676.2013.805404 (2014).

8 Arksey, H. & O'Malley, L. Scoping studies: towards a methodological framework. *International Journal of Social Research Methodology* **8**, 19-32 (2007).

9 Levac, D., Colquhoun, H. & O'Brien, K. Scoping studies: advancing the methodology. *Implementation Science* **5**, 69 (2010).

10 Adamson, J. Awareness and understanding of dementia in African/Caribbean and South Asian families. *Health Soc Care Community* **9**, 391-396 (2001).

11 Adamson, J. & Donovan, J. 'Normal disruption': South Asian and African/Caribbean relatives caring for an older family member in the UK. *Social Science and Medicine* **60**, 37-48 (2005).

12 Bhatnagar, K. & Frank, J. Psychiatric disorders in elderly from the Indian sub-continent living in Bradford. *International Journal of Geriatric Psychiatry* **12**, 907-912 (1997).

13 Bowes, A. & Wilkinson, H. 'We didn't know it would get that bad': South Asian experiences of dementia and the service response. *Health Soc Care Community* **11**, 387-396 (2003).

14 Giebel, C. *et al.* Perceptions of self-defined memory problems vary in south Asian minority older people who consult a GP and those who do not: A mixed-method pilot study. *International Journal of Geriatric Psychiatry* **31**, 379-387 (2016).

15 Haider, I. & Shah, A. A pilot study of behavioural and psychological signs and symptoms of dementia in patients of Indian sub-continent origin admitted to a dementia day hospital in the United Kingdom. *International Journal of Geriatric Psychiatry* **19**, 1195-1204 (2004).

16 Hailstone, J., Mukadam, N., Owen, T., Cooper, C. & Livingston, G. The development of attitudes of people from ethnic minorities to help-seeking for dementia (apend): A questionnaire to measure attitudes to help-seeking for dementia in people from south asian backgrounds in the uk. *International Journal of Geriatric Psychiatry Mar*, No Pagination Specified, doi:http://dx.doi.org/10.1002/gps.4462 (2016).

17 Jutlla, K. The impact of migration experiences and migration identities on the experiences of services and caring for a family member with dementia for Sikhs living in Wolverhampton,

For peer review only - http://bmjopen.bmj.com/site/about/guidelines.xhtml

UK. *Ageing & Society* **35**, 1032-1054, doi:http://dx.doi.org/10.1017/S0144686X14000658 (2015).
18 Kaur, H., Jutlla, K., Moreland, N. & Read, K. How a link nurse ensured equal treatment for people of Asian origin with dementia. *Nurs Times* **106**, 12-15 (2010).
19 La Fontaine, J., Ahuja, J., Bradbury, N. M., Phillips, S. & Oyebode, J. R. Understanding dementia amongst people in minority ethnic and cultural groups. *J Adv Nurs* **60**, 605-614 (2007).
20 Lawrence, V., Murray, J., Samsi, K. & Banerjee, S. Attitudes and support needs of Black Caribbean, south Asian and White British carers of people with dementia in the UK. *Br J Psychiatry* **193**, 240-246, doi:http://dx.doi.org/10.1192/bjp.bp.107.045187 (2008).
21 Lawrence, V., Samsi, K., Banerjee, S., Morgan, C. & Murray, J. Threat to valued elements of life: the experience of dementia across three ethnic groups. *Gerontologist* **51**, 39-50, doi:http://dx.doi.org/10.1093/geront/gnq073 (2011).
22 Lindesay, J. *et al.* The Mini-Mental State Examination (MMSE) in an elderly immigrant Gujarati population in the United Kingdom. *International Journal of Geriatric Psychiatry* **12**, 1155-1167 (1997).
23 Mackenzie, J. Stigma and dementia: East European and South Asian family carers negotiating stigma in the UK. *Dementia: The International Journal of Social Research and Practice* **5**, 233-247, doi:http://dx.doi.org/10.1177/1471301206062252 (2006).
24 Mukadam, N., Waugh, A., Cooper, C. & Livingston, G. What would encourage help-seeking for memory problems amongst South Asians? A qualitative study. *International Psychogeriatrics* **27**, S57-S58 (2015).
25 Odutoye, K. & Shah, A. The characteristics of Indian subcontinent origin elders newly referred to a psychogeriatric service. *International Journal of Geriatric Psychiatry* **14**, 446-453 (1999).
26 Purandare, N., Luthra, V., Swarbrick, C. & Bums, A. Knowledge of dementia among South Asian (Indian) older people in Manchester, UK. *International Journal of Geriatric Psychiatry* **22**, 777-781, doi:http://dx.doi.org/10.1002/gps.1740 (2007).
27 Rait, G. *et al.* Validating screening instruments for cognitive impairment in older South Asians in the United Kingdom. *International Journal of Geriatric Psychiatry* **15**, 54-62 (2000).
28 Seabrooke, V. & Milne, A. Early intervention in dementia care in an Asian community: Lessons from a dementia collaborative project. *Quality in Aging* **10**, 29-36 (2009).
29 Shah, A., Lindesay, J. & Jagger, C. Is the diagnosis of dementia stable over time among elderly immigrant Gujaratis in the United Kingdom (Leicester)? *International Journal of Geriatric Psychiatry* **13**, 440-444 (1998).
30 Shah, A. Difficulties experienced by a Gujarati geriatric psychiatrist in interviewing Gujarati elders in Gujarati. *International Journal of Geriatric Psychiatry* **14**, 1072-1074 (1999).
31 Uppal, G. K., Bonas, S. & Philpott, H. Understanding and awareness of dementia in the Sikh community. *Mental Health, Religion & Culture* **17**, 400-414, doi:http://dx.doi.org/10.1080/13674676.2013.816941 (2014).
32 Turner, S., Christie, A. & Haworth, E. South Asian and white older people and dementia: a qualitative study of knowledge and attitudes. *Diversity in Health and Social Care* **2**, 197-209 (2005).
33 Redelinghuys, J. & Shah, A. The characteristics of ethnic elders from the Indian subcontinent using a geriatric psychiatry service in West London. *Aging and Mental Health* **1**, 243-247 (1997).
34 Regan, J. L. Ethnic minority, young onset, rare dementia type, depression: A case study of a Muslim male accessing UK dementia health and social care services. *Dementia* **15**, 702-720, doi:doi:10.1177/1471301214534423 (2016).

For peer review only - http://bmjopen.bmj.com/site/about/guidelines.xhtml

35 Giebel, C. *et al.* Adaptation of the Barts explanatory model inventory to dementia understanding in South Asian ethnic minorities. *Aging and Mental Health* **20**, 594-602 (2016).
36 Moher, D., Liberati, A., Tetzlaff, J., Altman, D. & Group, T. P. Preferred reporting items for systematic reviews and meta-analysis: The PRISMA statmement *PLoS Medicine* **6**, e1000097, doi:10.1371/journal.pmed1000097 (2009).
37 Reisberg, B. *et al.* Behavioral symptoms in Alzheimer's disease: phenomenology and treatment. *Journal of Clinical Psychiatry* **48** (1987).
38 Graham, C., Ballard, C. & Sham, P. Carers' knowledge of dementia and their expressed concerns. *International Journal of Geriatric Psychiatry* **12**, 470 (1997).
39 Mathuranath, P., Nestor, P., Berrios, G., Rakowicz, W. & Hodges, J. A brief cognitive test battery to differentiate between Alzheimer's disease and frontotemporal dementia. *Neurology* **55**, 1613-1620 (2000).
40 Nasreddine, Z. *et al.* The Montreal Cognitive Assessment, MoCA: A brief screening tool for mild cognitive impairment *Journal of the American Geriatric Society* **53**, 695-699 (2005).

For peer review only

For peer review only - http://bmjopen.bmj.com/site/about/guidelines.xhtml

Figure 1: Flow of included studies [36]

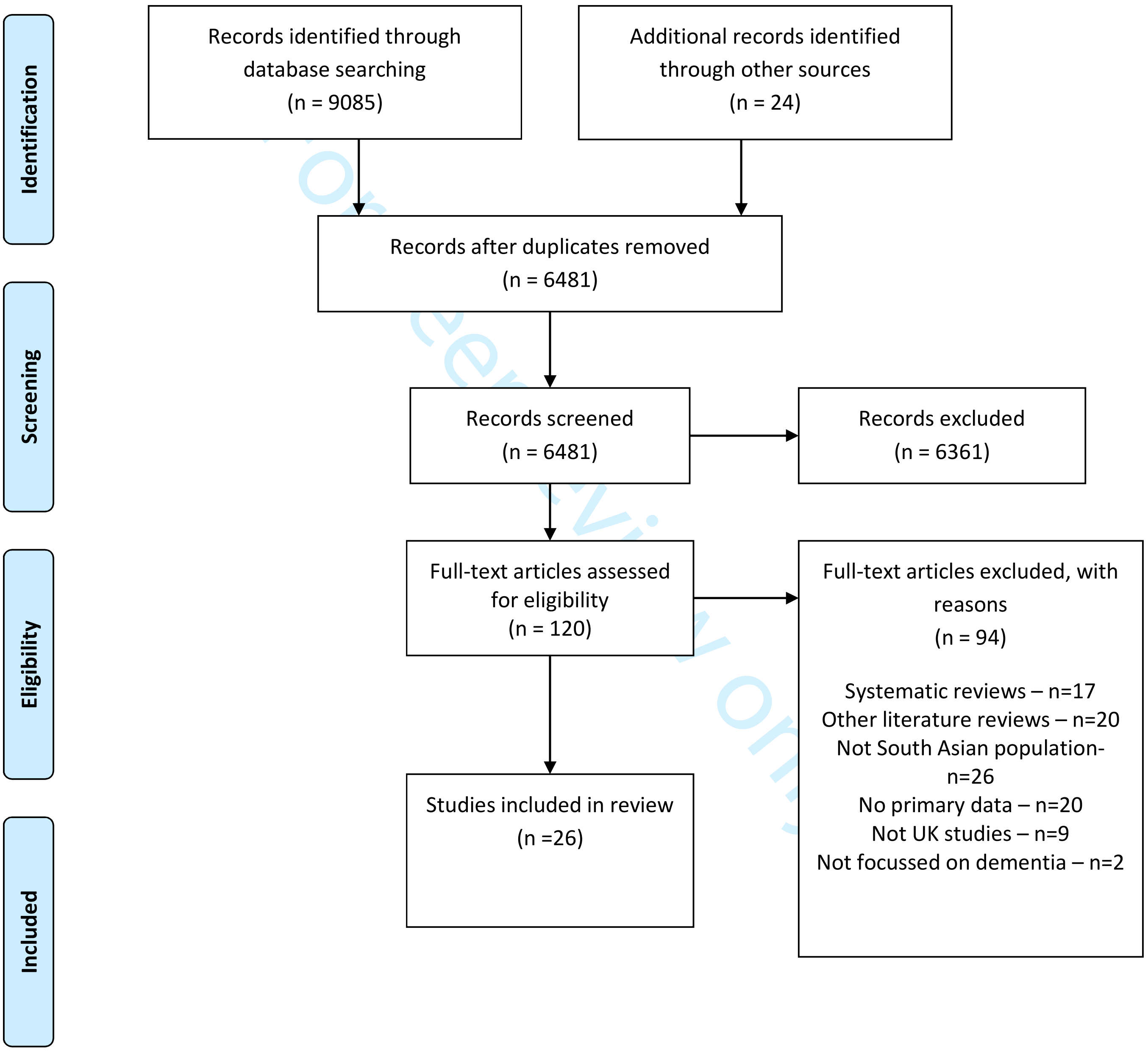

For peer review only - http://bmjopen.bmj.com/site/about/guidelines.xhtml

Table 1: Summary of characteristics and findings of included studies

Author	Year	Study Design	Total Sample Size	Sample	Setting	Summary of Main Findings
Adamson et al. [10]	2001	Qualitative	30	n=12 (40%) South Asian carers of people with dementia.	Healthcare services (carer support, day centres, psychiatric services)	Lack of knowledge of dementia found in South Asian group. South Asian participants talked about symptoms being a result of past actions in life and apportioning blame. They also believed that other physical conditions and their associated medications could cause dementia, such as antidepressants for depression.
Adamson et al. [11]	2005	Qualitative	36	n=15 (42%) South Asian carers of people with dementia	Community	South Asian participants talked about caring for family as a cultural norm and wider families were more likely to live together to facilitate this.
Bhatnagar et al. [12]	1997	Cross-sectional study	100	Aged 65-89y, from Indian subcontinent and living in Bradford	Community	Prevalence of dementia 4% as diagnosed by psychiatrist and 7% using Hindi translation of diagnostic measure (GMS-A).
Bowes et al. [13]	2003	Qualitative	11	11 interviews with carers<br>4 case studies of South Asian patients	Community	Carer interviews: demand for services, a need to develop awareness and knowledge in the community, and to promote a culturally sensitive response from services.<br>Case studies: negative experiences of dementia, poor quality of life, need for support, lack of access to appropriate services, little knowledge about dementia, isolation from both community and family life.
Giebel et al.[14]	2016	Mixed method pilot	33	3 groups - South Asian, over 60y:<br>without memory problems; memory problems not consulted GP; memory problems had consulted GP	Community	Those who had not consulted a GP often considered memory problems to be given by God and did not identify medical support as appropriate for them. Those who had attended a consultation with GP identified forgetfulness and loss of social meaning as symptoms of dementia

For peer review only - http://bmjopen.bmj.com/site/about/guidelines.xhtml

Author	Year	Study Design	Total Sample Size	Sample	Setting	Summary of Main Findings
Giebel et al.[35]	2016	Questionnaire validation	25	n=25 South Asian	Community	123 new perceptions around South Asian their understanding of dementia were identified. These were added to the BEMI-C to create a new checklist (BEMI-D).
Haider et al. [15]	2004	Pilot study	62	n=31 South Asian, aged 65-96y<br>n=31 White British, aged 65-90y	Day Hospital	South Asian participants score lower on the BEHAVE-AD phobia and anxiety subscale.<br>Alzheimer's disease associated with vascular dementia with affective disturbance.
Hailstone et al. [16]	2016	Questionnaire Validation	58	Mean age 60y<br>59% female (n=34)<br><br>1st and 2nd generation South Asians	Community	Strongest predictor of willingness to seek help for dementia was perceived social pressures from significant others.<br>Attitudes in the questionnaire predicted<br>77% of variance in willingness to seek help, but no relationship was found with dementia knowledge.
Jutlla et al [17]	2015	Qualitative	12	South Asian Sikhs caring for someone with dementia and living in Wolverhampton, UK	Community	Understandings participant's migration experiences and identities is important for understanding family carers experience of services when caring for someone with dementia.
Kaur et al. [18]	2010	Service Evaluation	N/A	An Asian Link Nurse working in Wolverhampton, UK	Community Mental Health Team	Having an Asian Link Nurse was vital in providing education about dementia for South Asian people.
La Fontaine et al. [19]	2007	Qualitative	49	South Asians aged 17-60y who were English, Hindi or Punjabi speaking	Community	Interviews highlighted that cognitive impairment was rarely mentioned when talking about ageing. Ageing was seen as a time of withdrawal and isolation. There was a sense of stigma and a lack of knowledge about mental health services l which leads to exclusion from these services.
Lawrence et al. [20]	2008	Qualitative	32	n=10 (31%) South Asian carers of people with	Community	South Asian carers possessed a traditional caregiver ideology, conceptualising caregiving as natural,

For peer review only - http://bmjopen.bmj.com/site/about/guidelines.xhtml

Author	Year	Study Design	Total Sample Size	Sample	Setting	Summary of Main Findings
				dementia		expected and virtuous. This informed their attitudes towards formal healthcare services.
Lawrence et al. [21]	2011	Qualitative	30	n=9 (30%) South Asian Aged 67-87y	Mental Health Services	Interviews highlighted that participants engaged in a process of appraisal where they assessed how much their condition affected valued elements of their life.
Lindesay et al. [22]	1997	Questionnaire Validation	1297	n=149 (11%) South Asian, Gujarati	General Practice	Mean MMSE scores were lower in the Gujarati group due to the effects of age, education and visial impairment.<br>The MMSE performed comparably in both groups as a screen for moderate to severe dementia but was less effective for Gujarati's with mild dementia.
Mackenzie et al. [23]	2006	Qualitative	21	n=16 (76%) South Asian carers of people with dementia	Community	In the South Asian group stigma was linked to religious and magical explanations for the onset of dementia which affected carers ability to access support.
Mukadam et al. [24]	2015	Qualitative	53	South Asians aged 18-83y	Community	Stigma around dementia was linked to ideas of 'madness' a lack of physical explanations and a lack of treatment.<br>Barriers to help seeking were that memory problems were an inevitable part of ageing. Denial of symptoms was evident in order to maintain position in the family and community, and due to fear of institutionalisation.
Odutoye et al. [25]	1999	Cross Sectional Study	242	n=29 (12%) South Asians newly referred to psychogeriatric unit between 1995-1997 aged 58-96y	Psycho-Geriatric Unit	South Asians were less likely to have dementia than White British elders (X^2 =5.05, 1df, P<0.03).
Purandare et al. [26]	2007	Cross Sectional	246	n=191 (78%) South Asian, mean age 72.4y	Community – day centre	Knowledge of dementia was poor in both South Asian and White British people. South Asians had less

For peer review only - http://bmjopen.bmj.com/site/about/guidelines.xhtml

Author	Year	Study Design	Total Sample Size	Sample	Setting	Summary of Main Findings
		Study		(sd 10.6).		knowledge about basic aspects of dementia (p<0.001) and the epidemiology of dementia (p<0.001) as compared to White British people.
Rait et al. [27]	2000	Validation of Screening Instrument	120	Community resident South Asians aged over 60y.<br>n=65 Gujarati speaking, mean age 70y (sd 6.8)<br>n=39 Pakistani group, mean age 68y (sd 6.0)	Community	Both modified screening tests (MMSE and AMT) had high sensitivity scores but ethnic background was found to influence the cut-off scores for these measures. The MMSE cut-off score was found to be significantly higher in the Pakistani group (>=27, sensitivity 100%, specificity 95%) compared with the Gujarati group (>=24, sensitivity 100%, specificity 77%).
Regan et al.[34]	2016	Case Study	N/A	Case study of a male Muslim patient with young onset fronto-temporal dementia	Dementia Services	Mostly negative experiences of accessing services and an inability to access support from either family or the religious community.<br>Services not equipped to support people with young onset dementia from an ethnic minority.
Seabrooke et al. [28]	2009	Service Pilot	4	South Asian patients aged 65-93y with memory problems	Primary Care	Inviting older Asian patients with memory problems to see a specially trained Asian nurse using a culturally appropriate information leaflet encouraged a small number of people to access the service.
Shah et al. [29]	1998	Longitudinal	11	Gujarati people over 65y living in Leicester, UK	Community	Seven of the 11 followed-up (64%). Diagnosis of dementia was reconfirmed in 6 out of 7 cases (86%) and there was evidence of further cognitive decline.
Shah et al. [30]	1999	Case Study – descriptive methodology	12	Gujarati patients (aged 65-90y) seen by Guajarati Psychogeriatrician.	Psycho-Geriatric Unit	n=4 (33%) with diagnosis of dementia. Difficulties interviewing Guajarati patients reported. Identifying cognitive signs and symptoms reported as most difficult. Few patients speak English and majority could not read or write.
Uppal et al. [31]	2014	Qualitative	28	Sikh participants over 18y	Community	Three key themes: awareness and interpretation of the characteristics of dementia; multiple perspectives of

For peer review only - http://bmjopen.bmj.com/site/about/guidelines.xhtml

Author	Year	Study Design	Total Sample Size	Sample	Setting	Summary of Main Findings
				Able to speak either Punjabi and/or English		the same symptoms; and causes of dementia.
Turner et al. [32]	2005	Qualitative	192	n=96 (50%) South Asian, aged 58-85y.	Community	South Asian people had less specific knowledge of dementia and believed that dementia was a normal part of ageing. Also less likely to think that medical treatment was available. Care was seen as provided by the family in the first instance.
Redelinghuys et al. [33]	1997	Cross Sectional Study	235	n=39 (17%) South Asians, aged 65-95y using a geriatric psychiatry service in South London.	Geriatric Psychiatry	n=6 (15%) of the South Asian group had dementia compared with n=43 (22%) of the White British elders. There were no differences found between the two groups in terms of use of health and social services.

GMS-A – Geriatric Mental State; BEHAVE-AS - Behavioural Pathology in Alzheimer's Disease Rating Scale; MMSE – Mini Mental State Examination; AMT – Abbreviated Mental Test; BEMI-D – Barts Explanatory Model Inventory Checklist; BEMI-D – Barts Explanatory Model Inventory Dementia.

For peer review only - http://bmjopen.bmj.com/site/about/guidelines.xhtml

Funding Statement

This work was supported by the National Institute for Health Research (NIHR) School for Primary Care Research (SPCR), grant number SPCR 300.

Competing Interest Statement

The authors declare they have no competing interests.

Author Contribution

AB and CK ran the search and screened papers. AB, CK, GDW, MP, WW identified and agreed eligible papers. AB wrote the paper and CK, GDW, MP, NM, WW edited and revised the paper for critical content.

Data Sharing Statement

No additional data are available.

For peer review only

For peer review only - http://bmjopen.bmj.com/site/about/guidelines.xhtml

Ovid Medline(R) 1946 to April Week 2 2016

View Saved

Search History (9)

	# ▲	Searches	Results	Type	
☐	1	Dementia/ or dementia.mp.	84164	Advanced	More
☐	2	Alzheimer Disease/ or Alzheimer* disease.mp.	101182	Advanced	More
☐	3	1 or 2	155203	Advanced	More
☐	4	ethnic*.mp.	126694	Advanced	More
☐	5	Asian.mp. or Asian Continental Ancestry Group/	89665	Advanced	More
☐	6	black.mp. or African Continental Ancestry Group/	104349	Advanced	More
☐	7	Ethnic Groups/ or multiethnic.mp.	54225	Advanced	More
☐	8	4 or 5 or 6 or 7	288052	Advanced	More

For peer review only

☐	9	3 and 8	2556	Advanced

For peer review only

BMJ Open

Dementia in UK South Asians: A scoping review of the literature

Journal:	*BMJ Open*
Manuscript ID	bmjopen-2017-020290.R1
Article Type:	Research
Date Submitted by the Author:	24-Jan-2018
Complete List of Authors:	Blakemore, Amy; University of Manchester, Division of Nursing, Social Work and Midwifery, School of Health Sciences, Manchester Academic Health Science Centre<br>Kenning, Cassandra; University of Manchester, Division of Population Health, Health Services Research and Primary Care, School of Health Sciences, Manchester Academic Health Science Centre<br>Mirza, Nadine; University of Manchester, Division of Population Health, Health Services Research and Primary Care, School of Health Sciences, Manchester Academic Health Science Centre<br>Daker-White, Gavin; University of Manchester, NIHR Greater Manchester Primary Care Patient Safety Translational Research Centre, Manchester Academic Health Science Centre<br>Panagioti, Maria; University of Manchester, Division of Population Health, Health Services Research and Primary Care, School of Health Sciences, Manchester Academic Health Science Centre<br>Waheed, Waquas; University of Manchester, Division of Population Health, Health Services Research and Primary Care, School of Health Sciences, Manchester Academic Health Science Centre
**Primary Subject Heading**:	Health services research
Secondary Subject Heading:	Neurology
Keywords:	Dementia < NEUROLOGY, South Asian, United Kingdom, Scoping Review

SCHOLARONE™
Manuscripts

Dementia in UK South Asians: A scoping review of the literature

Running head: Dementia in UK South Asians

Key Words: Dementia, United Kingdom, South Asian, Scoping Review

Authors:

Dr Amy Blakemore
Division of Nursing, Social Work and Midwifery, School of Health Sciences, Faculty of Biology, Medicine and Health, The University of Manchester, Manchester Academic Health Science Centre, Manchester, United Kingdom

Dr Cassandra Kenning
Division of Population Health, Health Services Research and Primary Care, School of Health Sciences, The University of Manchester, Manchester Academic Health Science Centre, Manchester, Manchester, United Kingdom

Miss Nadine Mirza
Division of Population Health, Health Services Research and Primary Care, School of Health Sciences, The University of Manchester, Manchester Academic Health Science Centre, Manchester, United Kingdom

Dr Gavin Daker-White
NIHR Greater Manchester Primary Care Patient Safety Translational Research Centre, The University of Manchester, Manchester Academic Health Science Centre, Manchester, United Kingdom

Dr Maria Panagioti
Division of Population Health, Health Services Research and Primary Care, School of Health Sciences, The University of Manchester, Manchester Academic Health Science Centre, Manchester, United Kingdom

Dr Waquas Waheed
Division of Population Health, Health Services Research and Primary Care, School of Health Sciences, The University of Manchester, Manchester Academic Health Science Centre, Manchester, United Kingdom

Corresponding Author
Dr Amy Blakemore, Division of Nursing, Social Work and Midwifery, Jean McFarlane Building, Oxford Road, The University of Manchester, Manchester M13 9PL
Tel: 0161 306 7846
Email: amy.blakemore@manchester.ac.uk

Word Count: 4892

For peer review only - http://bmjopen.bmj.com/site/about/guidelines.xhtml

Abstract (299 Words)

Objective

Over 850,000 people live with dementia in the United Kingdom (UK). A proportion of these people are South Asians, who make up over 5% of the total UK population. Little is known about the prevalence, experience, and treatment of dementia in the UK South Asian population. The aim of this scoping review is to identify dementia studies conducted in the UK South Asian population to highlight gaps in the literature which to be addressed in future research.

Method

Databases were systematically searched using a comprehensive search strategy to identify studies. A methodological framework for conducting scoping reviews was followed. An extraction form was developed to chart data and collate study characteristics and findings. Studies were then grouped into six categories: prevalence and characteristics; diagnosis validation and screening; knowledge, understanding and attitudes; help-seeking; experience of dementia; service organisation and delivery.

Results

A total of 6483 studies were identified,27 studies were eligible for inclusion in the scoping review. We found that studies of prevalence, diagnosis, and service organisation and delivery, in UK South Asians are limited. We did not find any clinical trials of culturally appropriate interventions for South Asians with dementia in the UK. The existing evidence comes from small scale service evaluations and case studies.

Conclusions

This is the first scoping review of the literature to identify priority areas for research to improve care for UK South Asians with dementia. Future research should first focus on developing and validating culturally appropriate diagnostic tools for UK South Asians and then conducting high quality epidemiological studies in order to accurately identify the prevalence of dementia in this group. The cultural adaptation of interventions for dementia

For peer review only

and testing in randomised controlled trials is also vital to ensure that there are appropriate treatments available for UK South Asians to access.

Strengths and Limitations of the Study

- The first scoping review of the literature to identify priority areas for future research and care of UK South Asians with dementia.
- Comprehensive and systematic search which was conducted in line with published guidelines for scoping reviews.
- A comprehensive and broad search strategy was developed however, a lack of standardisation around some key search terms could mean some studies were not identified.
- This review is not able to provide a definitive answer to any of the key themes discussed but its purpose is to map the literature and provide a much needed framework to guide future research directions for UK south Asians with dementia.

For peer review only

For peer review only - http://bmjopen.bmj.com/site/about/guidelines.xhtml

Introduction

As the global population ages the prevalence of dementia increases; there are an estimated 47 million people living with dementia worldwide and this figure is set to increase to over 75.6 million by 2030.[1,2] In the United Kingdom (UK) alone there are over 850,000 people living with dementia at a cost of £26 billion per year.[1] The rising prevalence of dementia and its associated cost and burden has generated an increased focus on the timely screening, diagnosis, and treatment of dementia.

Ethnic minority groups make up over 14% of the total UK population, many of whom are South Asian (Pakistan, India, and Bangladesh). In the 2001 census South Asians were 3.9% of the total UK population and by 2011 this figure had risen to 5.3%.[3] It is estimated that there are over 25,000 people from ethnic minority groups living with dementia in the UK.[4] However, the true prevalence of dementia within the UK South Asian community is yet to be established. As the overall prevalence of dementia in the UK increases so too will the prevalence amongst the growing population of UK South Asians. Increased prevalence places increased burden on health care services to understand the presentation of dementia in UK South Asians in order to identify, diagnose, and treat this population.

There are difficulties in identifying, diagnosing, and treating dementia in UK ethnic minority groups, including the South Asian population.[5,6] This is due to low levels of literacy, language barriers, and a lack of appropriately translated and culturally adapted screening and diagnostic tools for this ethnic group.[7] For example, in the South Asian community only 35% of older people (aged over 65 years) can speak English and only 21% can read and write English, with most relying instead on their first language which for many is Urdu.[3,8,9] This makes the completion of screening tools and diagnostic tests challenging. In addition to barriers of language and literacy there is a lack of awareness about dementia within the South Asian community. Many South Asian people view memory loss as a normal part of ageing or understand symptoms of dementia by religious belief.[7] This difference in the understanding and explanation of the presentation of dementia may result in reduced help-seeking.[7] Lack of help-seeking prohibits early intervention and treatment which is important to reduce the burden of dementia for patients, family carers, and the wider healthcare

For peer review only - http://bmjopen.bmj.com/site/about/guidelines.xhtml

system.[5,6,10] If we are to encourage UK South Asians to seek help early and improve our ability to engage this group with formal healthcare for dementia we need to provide culturally appropriate services for them to access. Currently there are a lack of both culturally appropriate services and accurately translated neuropsychological assessments for UK South Asians.[11]

In order to identify the scope of the literature on dementia in the UK South Asian population we have conducted a scoping review. Scoping reviews are designed to assess the nature and extent of the literature which is available on a topic.[12] The scoping process aims identify where there are gaps in the evidence around the prevalence, identification, diagnosis, and treatment of dementia among UK South Asians.[13] This will then allow recommendations to be made for future research; the results of which we hope will inform the identification, diagnosis, and treatment of dementia in UK South Asians.

For peer review only

Methods

To examine the literature on dementia in UK South Asians we conducted a scoping review in accordance with the methodological framework for scoping reviews published by Arskey and O'Malley (2007) and further developed by Levac et al. (2010).[14,15] The Arksey and O'Malley framework was found to be the most commonly used methodology for scoping reviews in a systematic review of scoping reviews.[13] The framework includes guidance on the following areas which are outlined in the sections below: identifying the research question, searching for relevant studies, selecting studies, charting the data, and collating and summarising results.

Inclusion criteria and exclusion criteria

Studies that had included UK South Asian dementia patients were eligible for inclusion, as well as those that had focused on family carers, and healthcare professionals working with dementia patients. We did not exclude studies based year of publication. Published dissertations were included but any unpublished dissertation was not. Systematic reviews and narrative literature reviews were not included but we hand searched the reference lists of all the relevant reviews which were returned in the search in order to identity additional primary studies for inclusion. Conference proceedings were not included.

We excluded studies that did not report on South Asian participants alone, as a comparator group, or where data could not be separated from data for participants of other ethnicities. We excluded studies that were not published in the English language.

Search strategy

Search terms were kept broad to identify the maximum number of studies which were eligible for inclusion. The search strategy was developed within the team to identify all studies relevant to ethnicity and dementia, this was then refined through hand checking to those which included UK South Asian participants. Search terms included: Dementia, Alzheimer* Disease; ethnic*, Asian, Black, African, minority, ethnic group, multi-ethnic. See additional material 1 for the full search strategy.

For peer review only - http://bmjopen.bmj.com/site/about/guidelines.xhtml

The search was conducted without a study design filter in order to retrieve studies using all methodologies, including: systematic reviews, qualitative studies, quantitative studies, case studies, and service evaluations.

The following databases were searched: Cochrane Register of Controlled Trials (CENTRAL), MEDLINE (OVID), PsycINFO (Ovid), Embase (Ovid), Cochrane database of systematic Reviews. The search was initially conducted in April 2016 and updated in June 2017. In addition to the database searches, we hand searched the reference lists of all relevant systematic reviews and literature reviews that were returned in the search to identify any additional papers for inclusion.

Screening and charting the data

Electronic search results were managed using EndNote X7 and Microsoft Excel. Titles and abstracts of all citations were first screened by author (AB), those that were not related to dementia and South Asians, or had been conducted outside of the UK, were discarded. The full text of all potentially eligible papers were then obtained and assessed against the inclusion criteria (AB and WW). Where the full text of papers was not available authors were contacted to request the paper. Any ambiguities about whether or not a study met the inclusion criteria were resolved by discussion at a meeting attended by all authors.

Studies that met the review criteria were charted using a data extraction sheet designed by AB and WW. Data were primarily extracted by AB and reviewed by WW. Data was extracted for the following domains: year of publication, study design, ethnicity, aim of study, study setting (community/primary care/hospital), eligibility criteria, participants (patient/carer/healthcare staff), type of dementia, age of participants, sample size, main findings.

Data synthesis

Once the pool of eligible studies was agreed we grouped them in to categories according to their primary focus (AB, CK, WW). These categories were defined as: prevalence and characteristics; service organisation and delivery; views and experiences; validation,

For peer review only - http://bmjopen.bmj.com/site/about/guidelines.xhtml

screening and diagnosis. All data are reported in a narrative format. As this is a scoping review there is no formal review of the quality of the included literature.[14,15]

Results

Description of included studies

The initial search identified 6483 studies after duplicates were removed. Screening of the titles and abstracts left 122 studies that met the criteria for full text review, 26 were then selected for inclusion.[16-41] To update the review the search was rerun in June 2017; 1 new paper was found to be eligible for inclusion, bringing the total number of included studies to 27.[42] Figure 1 shows the flow of study selection and numbers of excluded studies with reasons.[43]

Table 1 shows a summary of the findings for all 27 included studies. For the purpose of narrative review we grouped the studies into six categories: prevalence and characteristics; diagnosis validation and screening; knowledge, understanding and attitudes; help-seeking; experience of dementia; service organisation and delivery.

Prevalence and Characteristics

We identified five articles that had looked at either the prevalence or characteristics of dementia in UK South Asians.[18,21,31,39,41]

Bhatnagar (1997) looked at the prevalence of dementia in a community sample of people from the Indian subcontinent living in Bradford, UK.[18] Diagnostic interviews were conducted with 100 participants and the prevalence of dementia was reported as 4%. However, when tested using a Hindi translation of the Geriatric Mental State (GMS-A) which found an initial prevalence of 7%, the reliability of the diagnosis was poor. The authors suggest that this is due to a lack of cultural adaptation of the measure. For example some interviewees would have little understanding of western calendar months, a knowledge of which forms part of the screening measure.[18]

For peer review only - http://bmjopen.bmj.com/site/about/guidelines.xhtml

A cross-sectional study conducted in inner-city Liverpool also used the Geriatric Mental State interview to assess the prevalence of dementia in English speaking versus non English speaking Asian participants.[41] The authors do not use the term South Asian but rather Asian. However, we have included this paper as it is clear from the categorisation of other ethnic groups included in the study that Asian refers to participants from South Asia who make up 2% of the total study sample. The authors interviewed 12 Asian participants using the Geriatric Mental State Examination and found a prevalence of dementia of 9% amongst the English speaking sample.

Two articles reported characteristics of ethnic elders from the Indian subcontinent living in west London and attending a psychogeriatric service.[31,39] Both published reports were from the same sample population, the first consisting of cross sectional data from a census conducted in August 1995,[39] and the second a two year study of all new cases entering the service between August 1995 and July 1997.[31] Sample sizes were small with South Asians making up only 12%[31] and 17%[39] of the total number of recruited participants. Dementia was diagnosed by physicians and eligible patients were identified by case note review. Redelinghuys et al. 1997[39] found that only a small subsample of participants had dementia, with no significant difference found between those from the Indian subcontinent (n=6, 15%) and white British elders (n=43, 22%). However, Odutoye et al. 1999 [31] reported that over that time period there was a significant difference in the incidence of dementia with elders from the Indian subcontinent being less likely to have a dementia.

Haider et al. (2004) conducted a study of the behavioural and psychological signs and symptoms of dementia using the Behavioural Pathology in Alzheimer's disease Rating Scale (BEHAVE-AD) rating scale.[21,44] They compared a sample of physician diagnosed patients from the Indian subcontinent with a White British sample living in West London who had been referred to a day hospital for treatment. They did not find any major differences between the two groups.

In summary studies have attempted to identify the prevalence of dementia in South Asians living in the UK. Most have reported that there is no difference in the prevalence between South Asian and White British participants. However, sample sizes in these studies have

For peer review only - http://bmjopen.bmj.com/site/about/guidelines.xhtml

been unanimously small and the studies are over 14 years old, therefore it is difficult draw conclusions about the definitive prevalence. Several studies do not report sufficient detail on the methods and instruments by which dementia was diagnosed and if there was any translation or cultural adaptation. [21,31,39] Bhatnagar et al. (1997) noted that there is a lack of cultural adaptation of dementia measures for South Asians which may result in high false positive and false negative diagnoses which may result in unreliable prevalence rates.[18]

Diagnosis, Validation & Screening

Three papers were identified that discussed either the diagnosis of dementia in UK South Asians or validation of screening tools. Two studies were identified which had looked to validate the MMSE dementia screening instrument for use in the UK South Asian population. Lindesay et al. (1997) investigated whether a Gujarati version of the MMSE could be used as a screening instrument in a Gujarati population in Leicester.[28] The authors report that they followed the precedent of Ganguli et al. (1995) in developing this Gujarati version of the MMSE.[45] They initially selected the items using consensus, translation and back translation, pretesting and validation against psychiatric diagnosis.[28] Lindesay et al. (1997) report that the MMSE performed well in the identification of moderate to severe dementia but was less effective in detecting cases of mild dementia in the Gujarati group when compared to a group of White British participants. In a second study, using the same sample as Lindesay et al. (1997)[28], Shah et al. (1998) investigated the stability of dementia diagnosis over time.[35] They reported that the diagnosis of dementia was stable at follow-up over a period of more than 2 years (26-32 months) which indicates that the measure Gujarati MMSE has test-retest reliability in this population.

Rait et al. (2000) tested the MMSE in a population of South Asians, including Gujarati and Pakistani participants.[33] The authors state that, as there were no definitive guidelines available for translating questionnaires such as the MMSE, they engaged both academics and members of the South Asian community to assess the cultural sensitivity of the items in three groups. The items were translated by a professional translation group and then back-translated with the South Asian community group. They report that modifications were made to the MMSE which centred around education and literacy and the modified MMSE was tested in a pilot study.[46] They found high levels of sensitivity and specificity for the

For peer review only - http://bmjopen.bmj.com/site/about/guidelines.xhtml

For peer review only

MMSE but with a lower cut-off score for the identification of mild dementia in the South Asian group.

Findings indicate that the Guajarati version of the MMSE may be effective at identifying dementia, especially where the diagnosis is certain and therefore symptoms are moderate to severe. However, it may be less effective in identifying dementia where symptoms are mild or diagnosis is uncertain.

Knowledge, Understanding, and Attitudes

We identified six studies which had set out to identify knowledge, understanding and attitudes to dementia in the UK South Asian community. Knowledge about dementia in Indian (n=91) and White UK/Irish/European (n=55) people was assessed using the Dementia Knowledge Questionnaire (DKQ) by Purandare et al.[32] They found that both groups had poor knowledge of causes and symptoms, and Indian older people had significantly less 'basic knowledge' about dementia. Here the authors define 'basic knowledge as including knowledge of epidemiology, aetiology, and symptomatology as measured by the Dementia Knowledge Questionnaire (DKQ).[47] A further series of focus groups investigated general population knowledge of dementia with 28 Sikh participants.[37] Again they found poor knowledge of dementia and that Sikh participants placed greater emphasis on physical illnesses than disorders such as dementia. La Fontaine held focus groups with a population sample of 49 Punjabi Indian participants.[25] One of their key findings was the failure of participants to directly refer to dementia or even to use lay terms to discuss it which the authors conclude indicates a lack of recognition of dementia as a concept.

Turner et al. (2005) explored knowledge and attitudes to dementia in a qualitative community study of South Asian (n=96) and White older people (n=96) and also found reduced knowledge of dementia in the South Asian group. Furthermore, their findings highlighted differences in attitudes to dementia care, with the South Asian group reporting that care should be provided by family and friends.[38] In interviews with 10 South Asian family carers Lawrence et al. (2008) looked at traditional versus non-traditional care giver ideology.[26] The majority of South Asian carers possessed a traditional ideology in that they

For peer review only - http://bmjopen.bmj.com/site/about/guidelines.xhtml

saw care giving as natural, expected, and virtuous. However, where understanding of dementia is poor problems can occur within the family caring relationship due to beliefs about the causes of dementia in the South Asian community and the attribution of blame on to the person with dementia.[16]

Adamson et al. (2005) interviewed 15 South Asian carers living in five cities across the UK.[16] The study looked at the relationship between individual experiences, cultural factors and social structures within this minority population. Carers tended to interpret their caring roles as an expected part of their cultural heritage and a continuation of their family relationships. They also likened the experiences of this group to other chronic illness care and suggest that the understanding of the experiences of chronic care may be useful in understanding experiences in informal care for dementia.

In summary, several studies have explored the knowledge of dementia, and beliefs and attitudes to care in UK South Asians. All report that knowledge is poor and this group are often more focussed on physical illness rather than conditions such as dementia. This finding that this group do not identify with dementia as a concept further highlights the need for valid and reliable measures to be developed to identify and then diagnose dementia in this group.

Our findings show that care is preferred to be provided by family and within the community and therefore, UK South Asians may not access formal healthcare for dementia.
Giebel et al. (2016) have adapted the Barts Explanatory Model Inventory Checklist (BEMI-C) for use with South Asian ethnic minority groups (BEMI-Dementia).[42] They conducted 25 qualitative interviews with South Asians and identified 123 new perceptions around their understanding of dementia. These were added to the BEMI-C to create a new checklist (BEMI-D) to better assess the barriers to dementia service uptake for this group in the future.

For peer review only - http://bmjopen.bmj.com/site/about/guidelines.xhtml

Help-seeking

Five papers were identified that focused on different aspects of help-seeking for dementia in the UK South Asian population.

Two studies had looked at barriers to help-seeking for dementia. Firstly, in a community sample of English or Bengali speaking UK South Asians who did not have a diagnosis of dementia Mukadam et al (2015) identified the barriers and facilitators to help-seeking for memory problems.[30] They identified four main categories of barriers which interacted to prevent timely diagnosis of dementia: barriers to help-seeking for memory problems; the threshold for seeking help for memory problems; ways to overcome barriers to help-seeking; what features an educational resource should have. Secondly, Hailstone et al. (2016) devised and validated a theory of planned behaviour (Attitudes of People from Ethnic Minorities for Help-seeking for Dementia: APEND) questionnaire to predict medical help-seeking for dementia in UK South Asians.[22] They found that attitudes to dementia predicted 77% of variance in help-seeking with the strongest predictor being perceived social pressure.

Furthermore, there were two studies which had specifically identified religious explanations as barriers to help-seeking for dementia in South Asians. Mackenzie et al. (2006) interviewed 11 Pakistani and 5 Indian carers and found that stigma resulted from religious and magical beliefs around the causes of dementia and resulted in concealment from their own community and delays in help-seeking.[29] Giebel et al. (2016) looked at the differences in the perceptions of South Asians who do and do not consult a General Practitioner (GP) about dementia.[20] They found those who did not consult a GP were significantly more likely to consider memory problems as given by god, with the view that medical intervention was therefore inappropriate. In a case study of a Muslim, Pakistani patient accessing healthcare for dementia in the UK, Regan et al. (2016) further highlight the importance of understanding a person's religious community and its role in providing both support and reducing stigma and isolation.[40]

For peer review only - http://bmjopen.bmj.com/site/about/guidelines.xhtml

The included studies highlight that attitudes and beliefs about dementia can serve as barriers to accessing healthcare, in particular understanding dementia within a religious context can delay help seeking. However, religious communities can also play a vital role in supporting patients with dementia and in reducing stigma. This may be the case across other ethnic minority communities in the UK, not just South Asians. It is therefore important to learn more about the religious context in which people understand dementia and for healthcare providers to engage with local communities and religious leaders in order to work in partnership with them to increase support and reduce stigma.

Experiences of Dementia

We identified three articles that looked at understanding the experience of dementia for UK South Asians. Bowes et al 2003 explored the experiences of South Asian patients with dementia in Scotland.[19] Interviews were conducted with 11 health professionals, and four case studies were built up through multiple interviews with 4 people with dementia, as well as interviews with their family and carers. The authors reported overwhelmingly negative experiences of dementia and of health services from the case studies. From the interviews with health professionals the authors concluded that there was a need to develop and promote culturally sensitive services. Furthermore, the article by Jutlla (2015) looked at the experiences of 12 Sikh carers, caring for a family member with dementia, in Wolverhampton.[23] In particular they looked at the influences of migration experiences and migration identities. They found that knowledge a person's background and experiences is important for understanding how they then experience health services and caring roles. This is presented as an advocate, not just for culturally appropriate services, but also for person-centred dementia care.

Lawrence et al. (2011) looked at experiences of dementia in three ethnic groups, 11 Black Caribbean, 9 South Asian and 10 White British older people with dementia.[27] The paper reported a comparison in the personal experiences of the condition in the three groups. They reported similar themes across groups with a key finding being that patients assessed their condition by the degree to which it interfered with 'valued elements of life'. The

For peer review only - http://bmjopen.bmj.com/site/about/guidelines.xhtml

authors concluded that development of culturally sensitive approaches to care should promote roles, relationships and activities that the patient values.

Service Organisation and Delivery

We found three articles that discussed service organisation and delivery for South Asian patients with dementia in the UK. Firstly, in 1999 Shah, a Gujarati Psychogeriatrician, published a descriptive account of his own experiences working with 12 Gujarati patients in their language.[36] Four of the patients had a diagnosis of dementia. He identified particular problems in eliciting cognitive symptoms due to problems with the translation and the lack of culturally appropriate assessments.[36] Secondly, Kaur et al. (2010) writes about the role an Asian link nurse for Punjabi speaking people of Asian origin in a dementia service in Wolverhampton.[24] The authors reported success in providing appropriate and culturally sensitive help and information for healthcare professionals, voluntary services, and South Asian patients with dementia. Thirdly, Seabrooke and Milne (2009) discuss the Dementia Collaborative Project in North West Kent which aimed to raise awareness of memory problems in South Asians and facilitate access to screening and diagnosis.[34] The authors reported an increase in referrals to a specialist clinic for memory assessments, some of which were for South Asian patients, and an increase in health professional's knowledge of memory problems in South Asians.

For peer review only - http://bmjopen.bmj.com/site/about/guidelines.xhtml

Discussion

We have conducted the first scoping review of dementia in the UK South Asian population. We identified 27 studies which were focussed on the prevalence, diagnosis and screening, knowledge and attitudes, help seeking, experience, and service organisation and delivery for dementia in this group. Overall, this review highlights that research on dementia in UK South Asians is limited. Studies on prevalence are out-dated, we lack culturally adapted instruments to diagnose dementia, and community engagement work is in its infancy.

There have been few studies of the prevalence of dementia within UK South Asians and where studies have set out to identify prevalence, sample sizes have been small. Furthermore, prevalence studies are old, with the most recent being published in 2004 and therefore, the current prevalence of dementia in UK South Asians in unknown.[21] There is a need for large epidemiological studies of dementia in UK South Asians in order to confirm the prevalence nationally. There is also a need for studies embedded within epidemiological work that can explore the current experience, both quantitatively and qualitatively, of South Asians with dementia in the context of dementia healthcare. Future cohort studies of this nature should provide data grouped by ethnicity and that ethnic groups are well defined to allow results to be included in future reviews.

We found only two studies that aimed to validate the MMSE screening tool within the UK South Asian population.[28,33] These two studies limited South Asians to a screening process with no access to receiving a confirmed diagnosis. We did not identify any studies that had addressed the introduction and validation of a purely diagnostic assessment for UK South Asians, such as the Addenbrooke's Cognitive Examination Version III or the Motreal Cognitive Assessment.[48,49] This compromises current knowledge of dementia prevalence within in UK South Asians, as without a culturally appropriate, validated diagnostic assessment individuals cannot be diagnosed. The lack of a culturally adapted and validated diagnostic tool risks that UK South Asians who enter into the diagnostic process will receive higher rates of false positive or false negative scores. This invalidates the current diagnoses given to UK South Asians. We suggest that undertaking the cultural adaptation and

For peer review only - http://bmjopen.bmj.com/site/about/guidelines.xhtml

validation of diagnostic assessments according to published guidelines would improve the diagnostic process.[50]

Most of the existing research has been conducted in the community which reflects the fact that UK South Asians do not access formal healthcare services for memory problems. We need to develop culturally sensitive screening tools, diagnostic tests, and interventions whilst also engaging communities. It is important to develop interventions in parallel with community engagement to ensure that there are culturally appropriate services ready to be accessed if engagement is successfully increased.[7]

We were unable to identify a single clinical trial of an intervention for dementia in the UK South Asian population, either amongst patients or carers. This highlights a critical gap in ongoing dementia research and indicates a neglect of 5.3% of the UK population that identify as South Asian and calls into question whether it is appropriate to implement existing interventions for this ethnic group without any assessment of their feasibility, acceptability, and effectiveness.[3] This review has identified a number of different views and perceptions held about dementia by UK South Asians. Therefore, existing interventions need to be adapted, or new culturally sensitive interventions should be developed and trialled which can accommodate for these differences.. From our findings relating to service organisation and delivery we can see strategies emerging that may increase engagement from this population, such as the ethnic matching of staff and increasing engagement work with South Asian community. These strategies should now be considered when designing clinical trials to test culturally adapted interventions.

This review has highlighted factors which impact on our ability to conduct intervention trials in this population; these may include issues surrounding diagnosis and the lack of appropriately validated diagnostic instruments, as well as attitudes and beliefs about dementia which may affect recruitment. Much of the qualitative work identified in this review has highlighted the religious explanations by which the South Asian community often understand symptoms of dementia. Studies report that this causes a barrier to help seeking and will result in delayed treatment. Furthermore, due to religious explanatory models for dementia patients and family carers often perceive treatment as inappropriate. Culturally

For peer review only

For peer review only - http://bmjopen.bmj.com/site/about/guidelines.xhtml

sensitive community engagement work is needed to engage the South Asian community and encourage understanding of dementia. New interventions should also acknowledge and include the family approach to care which is seen as of paramount importance in the South Asian community.

This review is the first review to scope all of the literature on dementia in the UK South Asian population. We scoped the literature in line with the published guidelines for scoping reviews and conducted a systematic search of the literature.[13,15] However, it is possible that there are other studies that were not identified as part of this search; in part this could be due to a lack of standardisation of the terminologies used in the literature. One potential limitation of our scoping review is that we restricted our eligible studies to those conducted in the UK. Although narrow, our focus on UK South Asians allowed us to identify gaps in the literature for a group which may face specific challenges and barriers to accessing healthcare in the NHS. It would however, be beneficial to review studies of dementia in South Asians globally in order to identify key learning for development of any intervention.

The scoping review is not designed to provide a definitive answer to questions in any of the key themes discussed in this review because there are important differences across the included studies which were not extensively evaluated (e.g. quality, design, and population). Whilst this is consistent with published guidelines for scoping reviews it means that we are unable to identify any gaps in the literature which arise due to poor quality research.[13,14] The purpose of this review was to map the literature and to provide a useful framework to guide future research directions. The scoping review methodology is particularly suited to this aim as it provides a comprehensive account of current progress and challenges which can be used to develop future research and priorities to improve care for UK South Asians.[13]

Conclusion

A number of studies have been published on dementia in the UK South Asian population. However, studies of prevalence, diagnosis, and service organisation and delivery are limited. We found no clinical trials of culturally appropriate interventions for South Asians with

For peer review only - http://bmjopen.bmj.com/site/about/guidelines.xhtml

dementia in the UK. The existing evidence comes from small scale service evaluations and case studies.

Future research efforts should concentrate on developing and validating culturally appropriate diagnostic tools for UK South Asians. Epidemiological studies are needed to accurately identify the prevalence of dementia in this group. Cultural adaptation and clinical trials of appropriate culturally sensitive interventions are needed to run in parallel to diagnosis and community engagement work to ensure there are effectiveness and acceptable treatments for South Asians to access once identified with dementia.

For peer review only - http://bmjopen.bmj.com/site/about/guidelines.xhtml
For peer review only

Funding Statement

This paper represented independent research funded by the National Institute for Health Research (NIHR) School for Primary Care Research (SPCR), grant number SPCR 300. The views expressed are those of the author(s) and not necessarily those of the NHS, the National Institute for Health Research, or the Department of Health.

Competing Interest Statement

The authors declare they have no competing interests.

Author Contribution

AB and CK ran the search and screened papers. AB, CK, GDW, MP, WW identified and agreed eligible papers. AB wrote the paper and CK, GDW, MP, NM, WW edited and revised the paper for critical content.

Data Sharing Statement

No additional data are available.

For peer review only

For peer review only - http://bmjopen.bmj.com/site/about/guidelines.xhtml

Figure 1: Flow of Included Studies

For peer review only

For peer review only - http://bmjopen.bmj.com/site/about/guidelines.xhtml

References

1 Alzheimer's Disease Internataional. *Policy Brief for G8 Heads of Government. The Gloval Impact of Dementia 2013-2050*, <http://www.who.int/mental_health/neurology/dementia/dementia_thematicbrief_epidemiology.pdf> (2013).

2 World Health Organisation. *the Epidemiology and Impact of Dementia: Current state and future trends*, <http://www.who.int/mental_health/neurology/dementia/dementia_thematicbrief_epidemiology.pdf> (2015).

3 Office for National Statistics. *Ethnicity and National Identiity in England and Wales*, <https://www.ons.gov.uk/peoplepopulationandcommunity/culturalidentity/ethnicity/articles/ethnicityandnationalidentityinenglandandwales/2012-12-11> (2012).

4 All-Party Parliamentary Group on Dementia. *Dementia does not discriminate: The experiences of black, Asina and minority ethnic communities*, <https://www.alzheimers.org.uk/download/downloads/id/1857/appg_2013_bame_report.pdf> (2013).

5 Mukadam, N., Cooper, C. & Livingstone, G. A systematic review of ethnicity and pathways to care in dementia. *International Journal of Geriatric Psychiatry* **26**, 12-20 (2011).

6 Mukadam, N., Cooper, C. & Livingston, G. Improving access to dementia services for people from minority ethnic groups. *Current Opinion in Psychiatry* **26**, 409-414 (2013).

7 Kenning, C., Daker-White, G., Blakemore, A., Panagioti, M. & Waheed, W. Barriers and facilitators in accessing dementia care by ethnic minority groups: a meta-synthesis of qualitative studies. *BMC Psychiatry* **17**, 316, doi:10.1186/s12888-017-1474-0. (2017).

8 Khan, F. & Tadros, G. Complexity in cognitive assessment of elderly British minority ethnic groups: Cultural perspective. *Dementia* **13**, 467-482, doi:doi:10.1177/1471301213475539 (2014).

9 Lindesay, J., Jagger, C., Hibbett, M., Peet, S. & Moledina, F. Knowledge, update and availability of health and social services among asian gujurati and white elderly persons. *Ethnicity and Health* **2**, 59-69 (1997).

10 Cooper, C., Tandy, A., Balamurali, T. & Livingston, G. A systematic review and meta-analysis of ethnic differences in use of dementia treatment, care, and research. *American Journal of Geriatric Psychiatry* **18**, 193-203 (2010).

11 Regan, J. L. Redefining dementia care barriers for ethnic minorities: The religion-culture distinction. *Mental Health, Religion & Culture* **17**, 345-353, doi:http://dx.doi.org/10.1080/13674676.2013.805404 (2014).

12 Grant, M. & Booth, A. A typology of reviews: an analysis of 14 review types and associated methodologies. *Health Information and Libraries Journal* **26** (2009).

13 Pham, M. *et al.* A scoping review of scoping reviews: advancing the approach and enhancing the consistency. *Research Synthesis Methods* **5**, 371-385, doi:DOI: 10.1002/jrsm.1123 (2014).

14 Arksey, H. & O'Malley, L. Scoping studies: towards a methodological framework. *International Journal of Social Research Methodology* **8**, 19-32 (2007).

15 Levac, D., Colquhoun, H. & O'Brien, K. Scoping studies: advancing the methodology. *Implementation Science* **5**, 69 (2010).

16 Adamson, J. Awareness and understanding of dementia in African/Caribbean and South Asian families. *Health Soc Care Community* **9**, 391-396 (2001).

17 Adamson, J. & Donovan, J. 'Normal disruption': South Asian and African/Caribbean relatives caring for an older family member in the UK. *Social Science and Medicine* **60**, 37-48 (2005).

18 Bhatnagar, K. & Frank, J. Psychiatric disorders in elderly from the Indian sub-continent living in Bradford. *International Journal of Geriatric Psychiatry* **12**, 907-912 (1997).

19 Bowes, A. & Wilkinson, H. 'We didn't know it would get that bad': South Asian experiences of dementia and the service response. *Health Soc Care Community* **11**, 387-396 (2003).

For peer review only - http://bmjopen.bmj.com/site/about/guidelines.xhtml

20 Giebel, C. *et al.* Perceptions of self-defined memory problems vary in south Asian minority older people who consult a GP and those who do not: A mixed-method pilot study. *International Journal of Geriatric Psychiatry* **31**, 379-387 (2016).

21 Haider, I. & Shah, A. A pilot study of behavioural and psychological signs and symptoms of dementia in patients of Indian sub-continent origin admitted to a dementia day hospital in the United Kingdom. *International Journal of Geriatric Psychiatry* **19**, 1195-1204 (2004).

22 Hailstone, J., Mukadam, N., Owen, T., Cooper, C. & Livingston, G. The development of attitudes of people from ethnic minorities to help-seeking for dementia (apend): A questionnaire to measure attitudes to help-seeking for dementia in people from south asian backgrounds in the uk. *International Journal of Geriatric Psychiatry Mar*, No Pagination Specified, doi:http://dx.doi.org/10.1002/gps.4462 (2016).

23 Jutlla, K. The impact of migration experiences and migration identities on the experiences of services and caring for a family member with dementia for Sikhs living in Wolverhampton, UK. *Ageing & Society* **35**, 1032-1054, doi:http://dx.doi.org/10.1017/S0144686X14000658 (2015).

24 Kaur, H., Jutlla, K., Moreland, N. & Read, K. How a link nurse ensured equal treatment for people of Asian origin with dementia. *Nurs Times* **106**, 12-15 (2010).

25 La Fontaine, J., Ahuja, J., Bradbury, N. M., Phillips, S. & Oyebode, J. R. Understanding dementia amongst people in minority ethnic and cultural groups. *J Adv Nurs* **60**, 605-614 (2007).

26 Lawrence, V., Murray, J., Samsi, K. & Banerjee, S. Attitudes and support needs of Black Caribbean, south Asian and White British carers of people with dementia in the UK. *Br J Psychiatry* **193**, 240-246, doi:http://dx.doi.org/10.1192/bjp.bp.107.045187 (2008).

27 Lawrence, V., Samsi, K., Banerjee, S., Morgan, C. & Murray, J. Threat to valued elements of life: the experience of dementia across three ethnic groups. *Gerontologist* **51**, 39-50, doi:http://dx.doi.org/10.1093/geront/gnq073 (2011).

28 Lindesay, J. *et al.* The Mini-Mental State Examination (MMSE) in an elderly immigrant Gujarati population in the United Kingdom. *International Journal of Geriatric Psychiatry* **12**, 1155-1167 (1997).

29 Mackenzie, J. Stigma and dementia: East European and South Asian family carers negotiating stigma in the UK. *Dementia: The International Journal of Social Research and Practice* **5**, 233-247, doi:http://dx.doi.org/10.1177/1471301206062252 (2006).

30 Mukadam, N., Waugh, A., Cooper, C. & Livingston, G. What would encourage help-seeking for memory problems amongst South Asians? A qualitative study. *International Psychogeriatrics* **27**, S57-S58 (2015).

31 Odutoye, K. & Shah, A. The characteristics of Indian subcontinent origin elders newly referred to a psychogeriatric service. *International Journal of Geriatric Psychiatry* **14**, 446-453 (1999).

32 Purandare, N., Luthra, V., Swarbrick, C. & Bums, A. Knowledge of dementia among South Asian (Indian) older people in Manchester, UK. *International Journal of Geriatric Psychiatry* **22**, 777-781, doi:http://dx.doi.org/10.1002/gps.1740 (2007).

33 Rait, G. *et al.* Validating screening instruments for cognitive impairment in older South Asians in the United Kingdom. *International Journal of Geriatric Psychiatry* **15**, 54-62 (2000).

34 Seabrooke, V. & Milne, A. Early intervention in dementia care in an Asian community: Lessons from a dementia collaborative project. *Quality in Aging* **10**, 29-36 (2009).

35 Shah, A., Lindesay, J. & Jagger, C. Is the diagnosis of dementia stable over time among elderly immigrant Gujaratis in the United Kingdom (Leicester)? *International Journal of Geriatric Psychiatry* **13**, 440-444 (1998).

36 Shah, A. Difficulties experienced by a Gujarati geriatric psychiatrist in interviewing Gujarati elders in Gujarati. *International Journal of Geriatric Psychiatry* **14**, 1072-1074 (1999).

For peer review only

For peer review only - http://bmjopen.bmj.com/site/about/guidelines.xhtml

37 Uppal, G. K., Bonas, S. & Philpott, H. Understanding and awareness of dementia in the Sikh community. *Mental Health, Religion & Culture* **17**, 400-414, doi:http://dx.doi.org/10.1080/13674676.2013.816941 (2014).

38 Turner, S., Christie, A. & Haworth, E. South Asian and white older people and dementia: a qualitative study of knowledge and attitudes. *Diversity in Health and Social Care* **2**, 197-209 (2005).

39 Redelinghuys, J. & Shah, A. The characteristics of ethnic elders from the Indian subcontinent using a geriatric psychiatry service in West London. *Aging and Mental Health* **1**, 243-247 (1997).

40 Regan, J. L. Ethnic minority, young onset, rare dementia type, depression: A case study of a Muslim male accessing UK dementia health and social care services. *Dementia* **15**, 702-720, doi:doi:10.1177/1471301214534423 (2016).

41 McCracken, C. *et al.* Prevalence of dementia and depression among elderly people in Black and ethnic minorities. *The British Journal of Psychiatry* **171**, 269-273 (1997).

42 Giebel, C. *et al.* Adaptation of the Barts explanatory model inventory to dementia understanding in South Asian ethnic minorities. *Aging and Mental Health* **20**, 594-602 (2016).

43 Moher, D., Liberati, A., Tetzlaff, J., Altman, D. & Group, T. P. Preferred reporting items for systematic reviews and meta-analysis: The PRISMA statmement *PLoS Medicine* **6**, e1000097, doi:10.1371/journal.pmed1000097 (2009).

44 Reisberg, B. *et al.* Behavioral symptoms in Alzheimer's disease: phenomenology and treatment. *Journal of Clinical Psychiatry* **48** (1987).

45 Ganguli, M. *et al.* A hindi version of the MMSE: The development of a cognitive screening instrument for a largely illiterate rural elderly population in india. *International Journal of Geriatric Psychiatry* **10**, 367-377 (1995).

46 Rait, G., Morley, M., Lambat, I. & Burns, A. Modification of brief cognitive assessments for use with elderly people from the South Asian subcontinent. *Ageing and Mental Health* **1**, 356-363 (1997).

47 Graham, C., Ballard, C. & Sham, P. Carers' knowledge of dementia and their expressed concerns. *International Journal of Geriatric Psychiatry* **12**, 470 (1997).

48 Mathuranath, P., Nestor, P., Berrios, G., Rakowicz, W. & Hodges, J. A brief cognitive test battery to differentiate between Alzheimer's disease and frontotemporal dementia. *Neurology* **55**, 1613-1620 (2000).

49 Nasreddine, Z. *et al.* The Montreal Cognitive Assessment, MoCA: A brief screening tool for mild cognitive impairment *Journal of the American Geriatric Society* **53**, 695-699 (2005).

50 Mirza, N., Panagioti, M., Waheed, M. & Waheed, W. Reporting of the translation and cultural adaptation procedures of the Addenbrooke's Cognitive Examonation version III (ACE-III) and its predecessors: a systematic review. *BMC Medical Research Methodology* **17**, 141, doi:https://doi.org/10.1186/s12874-017-0413-6 (2017).

For peer review only

Table 1: Summary of characteristics and findings of included studies

Author	Year	Study Design	Total Sample Size	Sample	Setting	Summary of Main Findings
Adamson et al. [16]	2001	Qualitative	30	n=12 (40%) South Asian carers of people with dementia.	Healthcare services (carer support, day centres, psychiatric services)	Lack of knowledge of dementia found in South Asian group. South Asian participants talked about symptoms being a result of past actions in life and apportioning blame. They also believed that other physical conditions and their associated medications could cause dementia, such as antidepressants for depression.
Adamson et al. [17]	2005	Qualitative	36	n=15 (42%) South Asian carers of people with dementia	Community	South Asian participants talked about caring for family as a cultural norm and wider families were more likely to live together to facilitate this.
Bhatnagar et al. [18]	1997	Cross-sectional study	100	Aged 65-89y, from Indian subcontinent and living in Bradford	Community	Prevalence of dementia 4% as diagnosed by psychiatrist and 7% using Hindi translation of diagnostic measure (GMS-A).
Bowes et al. [19]	2003	Qualitative	11	11 interviews with carers<br>4 case studies of South Asian patients	Community	Carer interviews: demand for services, a need to develop awareness and knowledge in the community, and to promote a culturally sensitive response from services.<br>Case studies: negative experiences of dementia, poor quality of life, need for support, lack of access to appropriate services, little knowledge about dementia, isolation from both community and family life.
Giebel et al.[20]	2016	Mixed method pilot	33	3 groups – South Asian, over 60y:<br>without memory problems; memory problems not consulted GP; memory problems had consulted GP	Community	Those who had not consulted a GP often considered memory problems to be given by God and did not identify medical support as appropriate for them. Those who had attended a consultation with GP identified forgetfulness and loss of social meaning as symptoms of dementia

For peer review only - http://bmjopen.bmj.com/site/about/guidelines.xhtml

Author	Year	Study Design	Total Sample Size	Sample	Setting	Summary of Main Findings
Giebel et al.[42]	2016	Questionnaire validation	25	n=25 South Asian	Community	123 new perceptions around South Asian their understanding of dementia were identified. These were added to the BEMI-C to create a new checklist (BEMI-D).
Haider et al. [21]	2004	Pilot study	62	n=31 South Asian, aged 65-96y<br>n=31 White British, aged 65-90y	Day Hospital	South Asian participants score lower on the BEHAVE-AD phobia and anxiety subscale.<br>Alzheimer's disease associated with vascular dementia with affective disturbance.
Hailstone et al. [22]	2016	Questionnaire Validation	58	Mean age 60y<br>59% female (n=34)<br><br>1st and 2nd generation South Asians	Community	Strongest predictor of willingness to seek help for dementia was perceived social pressures from significant others.<br>Attitudes in the questionnaire predicted<br>77% of variance in willingness to seek help, but no relationship was found with dementia knowledge.
Jutlla et al [23]	2015	Qualitative	12	South Asian Sikhs caring for someone with dementia and living in Wolverhampton, UK	Community	Understandings participant's migration experiences and identities is important for understanding family carers experience of services when caring for someone with dementia.
Kaur et al. [24]	2010	Service Evaluation	N/A	An Asian Link Nurse working in Wolverhampton, UK	Community Mental Health Team	Having an Asian Link Nurse was vital in providing education about dementia for South Asian people.
La Fontaine et al. [25]	2007	Qualitative	49	South Asians aged 17-60y who were English, Hindi or Punjabi speaking	Community	Interviews highlighted that cognitive impairment was rarely mentioned when talking about ageing. Ageing was seen as a time of withdrawal and isolation. There was a sense of stigma and a lack of knowledge about mental health services I which leads to exclusion from these services.

For peer review only - http://bmjopen.bmj.com/site/about/guidelines.xhtml

Author	Year	Study Design	Total Sample Size	Sample	Setting	Summary of Main Findings
Lawrence et al. [26]	2008	Qualitative	32	n=10 (31%) South Asian carers of people with dementia	Community	South Asian carers possessed a traditional caregiver ideology, conceptualising caregiving as natural, expected and virtuous. This informed their attitudes towards formal healthcare services.
Lawrence et al. [27]	2011	Qualitative	30	n=9 (30%) South Asian Aged 67-87y	Mental Health Services	Interviews highlighted that participants engaged in a process of appraisal where they assessed how much their condition affected valued elements of their life.
Lindesay et al. [28]	1997	Questionnaire Validation	1297	n=149 (11%) South Asian, Gujarati	General Practice	Mean MMSE scores were lower in the Gujarati group due to the effects of age, education and visial impairment.<br>The MMSE performed comparably in both groups as a screen for moderate to severe dementia but was less effective for Gujarati's with mild dementia.
Mackenzie et al. [29]	2006	Qualitative	21	n=16 (76%) South Asian carers of people with dementia	Community	In the South Asian group stigma was linked to religious and magical explanations for the onset of dementia which affected the ability of carers to access support.
McCracken et al. [41]	1997	Cross Sectional Study	579	n=12 (2%) Asian Aged over 65 years	Community	Prevalence of dementia 9% among English speaking Asian participants.
Mukadam et al. [30]	2015	Qualitative	53	South Asians aged 18-83y	Community	Stigma around dementia was linked to ideas of 'madness' a lack of physical explanations and a lack of treatment.<br>Barriers to help seeking were that memory problems were an inevitable part of ageing. Denial of symptoms was evident in order to maintain position in the family and community, and due to fear of institutionalisation.
Odutoye et al.	1999	Cross	242	n=29 (12%) South	Psycho-	South Asians were less likely to have dementia than

For peer review only - http://bmjopen.bmj.com/site/about/guidelines.xhtml

Author	Year	Study Design	Total Sample Size	Sample	Setting	Summary of Main Findings
[31]		Sectional Study		Asians newly referred to psychogeriatric unit between 1995-1997 aged 58-96y	Geriatric Unit	White British elders (X^2 =5.05, 1df, P<0.03).
Purandare et al. [32]	2007	Cross Sectional Study	246	n=191 (78%) South Asian, mean age 72.4y (sd 10.6).	Community – day centre	Knowledge of dementia was poor in both South Asian and White British people. South Asians had less knowledge about basic aspects of dementia (p<0.001) and the epidemiology of dementia (p<0.001) as compared to White British people.
Rait et al. [33]	2000	Validation of Screening Instrument	120	Community resident South Asians aged over 60y.<br>n=65 Gujarati speaking, mean age 70y (sd 6.8)<br>n=39 Pakistani group, mean age 68y (sd 6.0)	Community	Both modified screening tests (MMSE and AMT) had high sensitivity scores but ethnic background was found to influence the cut-off scores for these measures. The MMSE cut-off score was found to be significantly higher in the Pakistani group (>=27, sensitivity 100%, specificity 95%) compared with the Gujarati group (>=24, sensitivity 100%, specificity 77%).
Regan et al.[40]	2016	Case Study	N/A	Case study of a male Muslim patient with young onset fronto-temporal dementia	Dementia Services	Mostly negative experiences of accessing services and an inability to access support from either family or the religious community.<br>Services not equipped to support people with young onset dementia from an ethnic minority.
Seabrooke et al. [34]	2009	Service Pilot	4	South Asian patients aged 65-93y with memory problems	Primary Care	Inviting older Asian patients with memory problems to see a specially trained Asian nurse using a culturally appropriate information leaflet encouraged a small number of people to access the service.
Shah et al. [35]	1998	Longitudinal	11	Gujarati people over 65y living in Leicester, UK	Community	Seven of the 11 followed-up (64%). Diagnosis of dementia was reconfirmed in 6 out of 7 cases (86%) and there was evidence of further cognitive decline.
Shah et al. [36]	1999	Case Study –	12	Gujarati patients (aged	Psycho-	n=4 (33%) with diagnosis of dementia. Difficulties

For peer review only - http://bmjopen.bmj.com/site/about/guidelines.xhtml

Author	Year	Study Design	Total Sample Size	Sample	Setting	Summary of Main Findings
		descriptive methodology		65-90y) seen by Guajarati Psychogeriatrician.	Geriatric Unit	interviewing Guajarati patients reported. Identifying cognitive signs and symptoms reported as most difficult. Few patients speak English and majority could not read or write.
Uppal et al. [37]	2014	Qualitative	28	Sikh participants over 18y<br>Able to speak either Punjabi and/or English	Community	Three key themes: awareness and interpretation of the characteristics of dementia; multiple perspectives of the same symptoms; and causes of dementia.
Turner et al. [38]	2005	Qualitative	192	n=96 (50%) South Asian, aged 58-85y.	Community	South Asian people had less specific knowledge of dementia and believed that dementia was a normal part of ageing. Also less likely to think that medical treatment was available. Care was seen as provided by the family in the first instance.
Redelinghuys et al. [39]	1997	Cross Sectional Study	235	n=39 (17%) South Asians, aged 65-95y using a geriatric psychiatry service in South London.	Geriatric Psychiatry	n=6 (15%) of the South Asian group had dementia compared with n=43 (22%) of the White British elders. There were no differences found between the two groups in terms of use of health and social services.

GMS-A – Geriatric Mental State; BEHAVE-AS - Behavioural Pathology in Alzheimer's Disease Rating Scale; MMSE – Mini Mental State Examination; AMT – Abbreviated Mental Test; BEMI-D – Barts Explanatory Model Inventory Checklist; BEMI-D – Barts Explanatory Model Inventory Dementia.

For peer review only - http://bmjopen.bmj.com/site/about/guidelines.xhtml

For peer review only

For peer review only - http://bmjopen.bmj.com/site/about/guidelines.xhtml

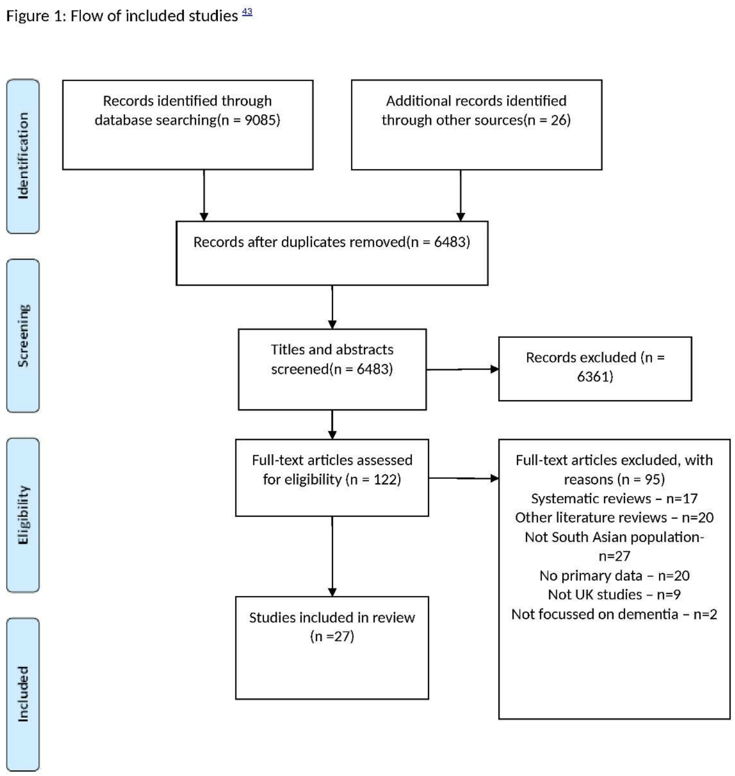

Figure 1: Flow of included studies

209x297mm (300 x 300 DPI)

Ovid Medline(R) 1946 to April Week 2 2016

View Saved

Search History (9)

# ▲	Searches	Results	Type	
1	Dementia/ or dementia.mp.	84164	Advanced	More
2	Alzheimer Disease/ or Alzheimer* disease.mp.	101182	Advanced	More
3	1 or 2	155203	Advanced	More
4	ethnic*.mp.	126694	Advanced	More
5	Asian.mp. or Asian Continental Ancestry Group/	89665	Advanced	More
6	black.mp. or African Continental Ancestry Group/	104349	Advanced	More
7	Ethnic Groups/ or multiethnic.mp.	54225	Advanced	More
8	4 or 5 or 6 or 7	288052	Advanced	More

☐	9	3 and 8	2556	Advanced

For peer review only
